# Mequindox Induced Genotoxicity and Carcinogenicity in Mice

**DOI:** 10.3389/fphar.2018.00361

**Published:** 2018-04-10

**Authors:** Qianying Liu, Zhixin Lei, Qin Wu, Deyu Huang, Shuyu Xie, Xu Wang, Yuanhu Pan, Zonghui Yuan

**Affiliations:** ^1^National Reference Laboratory of Veterinary Drug Residues (HZAU) and MAO Key Laboratory for Detection of Veterinary Drug Residues, Huazhong Agricultural University, Wuhan, China; ^2^Key Laboratory of Preventive Veterinary Medicine in Hubei Province, Huazhong Agricultural University, Wuhan, China; ^3^MOA Laboratory for Risk Assessment of Quality and Safety of Livestock and Poultry Products, Huazhong Agricultural University, Wuhan, China; ^4^Hubei Collaborative Innovation Center for Animal Nutrition and Feed Safety, Wuhan, China

**Keywords:** mequindox, quinoxaline, carcinogenicity, genotoxicity, KM mice

## Abstract

Mequindox (MEQ), acting as an inhibitor of deoxyribonucleic acid (DNA) synthesis, is a synthetic heterocyclic *N*-oxides. To investigate the potential carcinogenicity of MEQ, four groups of Kun-Ming (KM) mice (50 mice/sex/group) were fed with diets containing MEQ (0, 25, 55, and 110 mg/kg) for one and a half years. The result showed adverse effects on body weights, feed consumption, hematology, serum chemistry, organ weights, relative organ weights, and incidence of tumors during most of the study period. Treatment-related changes in hematology, serum chemistry, relative weights and histopathological examinations revealed that the hematological system, liver, kidneys, and adrenal glands, as well as the developmental and reproductive system, were the main targets after MEQ administration. Additionally, MEQ significantly increased the frequency of micronucleated normochromatic erythrocytes in bone marrow cells of mice. Furthermore, MEQ increased the incidence of tumors, including mammary fibroadenoma, breast cancer, corticosuprarenaloma, haemangiomas, hepatocarcinoma, and pulmonary adenoma. Interestingly, the higher incidence of tumors was noted in M25 mg/kg group, the lowest dietary concentration tested, which was equivalent to approximately 2.25 and 1.72 mg/kg b.w./day in females and males, respectively. It was assumed that the lower toxicity might be a reason for its higher tumor incidence in M25 mg/kg group. This finding suggests a potential relationships among the dose, general toxicity and carcinogenicity *in vivo*, and further study is required to reveal this relationship. In conclusion, the present study demonstrates that MEQ is a genotoxic carcinogen in KM mice.

## Introduction

**FIGURE 1:**
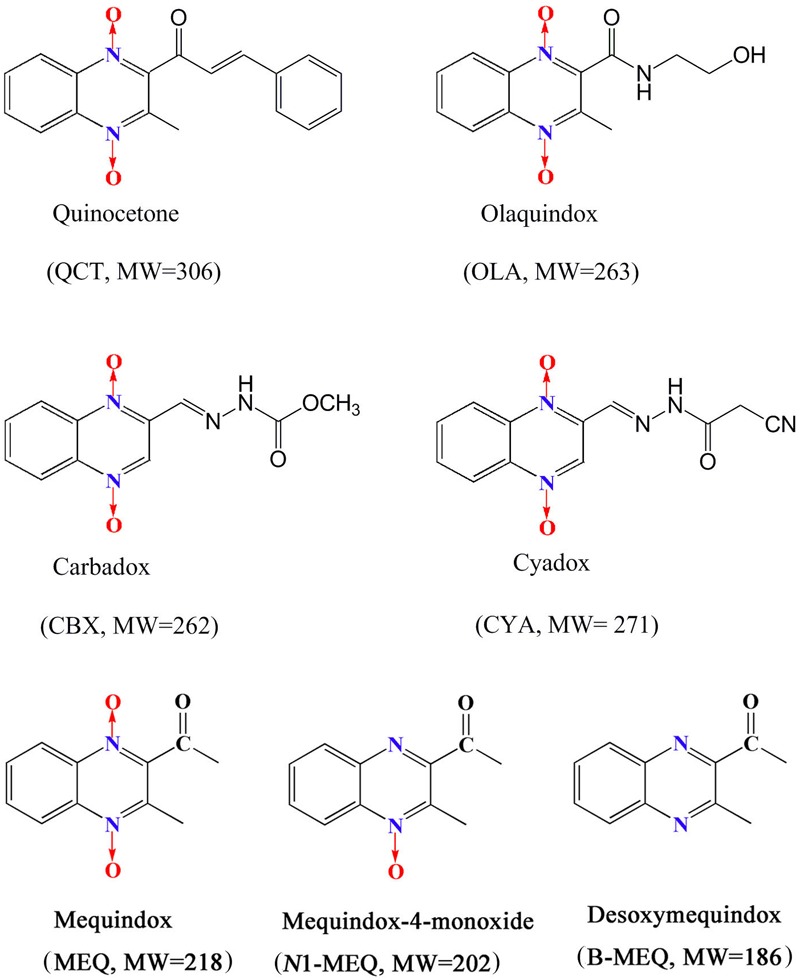
The chemical structures of Quinoxaline-di-*N*-oxides.

Quinoxaline-di-*N*-oxides (QdNOs), consisting of one or two acyclic chain moiety combined with quinoxaline ring, are a great family with the wide range of biological properties, including antibacterial, anti-candida, antitubercular, anticancer, antiprotozoal and growth promoting activities (Carta et al., 2005; Cheng et al., 2015; Vicente et al., 2009; Wang et al., 2011a, 2016b; Liu et al., 2017a). Four QdNOs, carbadox (CBX), olaquindox (OLA), quinocetone (QCT), and cyadox (CYA) (**Figure 1**), are used as growth promoters in livestock and poultry farming because of their strong antimicrobial activities (Liu et al., 2016, 2017a,b,d). Mequindox ([3-methyl-2-acetyl] quinoxaline-1,4-dioxide, C_11_H_10_N_2_O_3_; MEQ) (**Figure 1**), a relative new compound in QdNOs, is a synthetic antibacterial agent with strong inhibitory effect against both Gram-positive and negative bacteria (Ihsan et al., 2010, 2011, 2013a).

Mequindox was developed by the Lanzhou Institute of Animal Husbandry and Veterinary Drugs at the Chinese Academy of Agricultural Sciences. It was widely used in the food-producing animals in China due to its efficacious in the treatment of clinical infections caused by *Treponeme, Pasteurella, E. coli, Staphylococcus aureus*, and *Salmonella* sp. (Ihsan et al., 2011, 2013a; Ding et al., 2012; Liu et al., 2017c,b). However, the use of MEQ as animal feed additive or antimicrobial agent has raised serious health hazard effects. It was reported that MEQ had adverse effects on pigs and chicken in clinical use (Chun, 2005; Hongxia et al., 2005; Ihsan, 2011). The previous studies revealed that long-term MEQ treatment induced adrenal (Huang et al., 2009), endocrine and reproductive system toxicity in Wistar rats (Ihsan et al., 2011). In subchronic 90-day administration of MEQ (55, 110, and 275 mg/kg) to Wistar rats, MEQ (275 mg/kg) led to a reduction in body weight, and caused hepatic and adrenal histological changes (Ihsan et al., 2010). MEQ induced oxidative stress in liver and kidney in rats after exposure to MEQ (25, 55, 110, and 275 mg/kg) for 180 days (Huang et al., 2009, 2010b). Recently, we also found that MEQ produced toxicity in liver and testis of KM mice at a dose level of 25, 55, and 110 mg/kg diet (Liu et al., 2017b,c,d).

The relationship between genotoxicity and carcinogenicity was well documented in the extensive reviews (Brambilla and Martelli, 2007, 2009; Brambilla et al., 2010; Liu et al., 2017e). Indeed, due to the high prediction of genotoxicity for carcinogenicity, it was forbidden to use compounds with proven genotoxicity in humans except for some special drugs (e.g., anticancer) that can interact with DNA (ICH, 2012; Liu et al., 2017e). CBX was proved to be mutagenicity and carcinogenicity with developmental and reproductive toxicities (World Health Organization [WHO], 1991a,b; Ihsan et al., 2013a,b; Liu et al., 2016), and therefore, it was prohibited in food-producing animals by the Health Department of Canada and Commission of the European Community (Wu et al., 2007; Liu et al., 2017a). Bidesoxy-carbadox (B-CBX), a metabolite of CBX, was confirmed to be the genotoxic carcinogen by JECFA (JECFA, 2003). A lot of evidences indicated that MEQ caused chromosomal aberrations in V79 cells and invoked micronucleus formation in mice (Wang et al., 2011a,c; Ihsan et al., 2013a; Liu et al., 2016). Compared with CBX, a higher mutagenicity of MEQ to mammalian cells was found in *in vitro* and *in vivo* short-term tests (Ihsan et al., 2013a). Recently, it was revealed that two primary metabolites of MEQ, *N*1-desoxymequindox (*N*1-MEQ) and B-MEQ (**Figure 1**), gave positive results in a set of four different genotoxicity tests (Liu et al., 2016). Additionally, it was reported that the genotoxicity of B-MEQ was higher than B-CBX, which was consistent with their prototype drugs (MEQ > CBX) (Liu et al., 2016).

As an important component of preclinical safety package for MEQ, many studies have been conducted to evaluate its pharmacokinetics, reproductive toxicity, genotoxic toxicity and other general toxicity. These studies indicated that MEQ might be a potential genotoxic carcinogen. Since MEQ is intended for chronic use and its structural analogs like CBX and OLA are considered as carcinogens, the carcinogenicity study of MEQ is urgently needed to provide a complete toxicity spectrum of MEQ. In the present study, the carcinogenicity dietary feeding study of MEQ in KM mice was performed in accordance with the FDA Redbook 2000 (FDA, 2000a,b,c), China GB guidelines (Chinese Standard GB15193.17, 2003) and the OECD Guidelines (OECD, 2009).

## Materials and Methods

### Test Material

Mequindox (C_11_H_10_N_2_O_3_, molecular weight 218.21 g/mol, CAS No: 60875-16-3, purity 99.5%) was supplied by the Institute of Veterinary Pharmaceuticals, Huazhong Agricultural University (Wuhan, China). All other reagents were of analytical grade.

### Animals and Diet Preparation

Four hundred SPF KM mice (6–7 weeks old) weighting 25–36 g, were obtained from Center of Laboratory Animals of Hubei Province (Wuhan, China). For each sex, the individual body weights were within ±20% of the average. The study was approved by the Ethical Committee of the Faculty of Veterinary Medicine (Huazhong Agricultural University). The use of animals was in compliance with NIH Publication “The Development of Science Based Guidelines for Laboratory Animal Care” (NRC, 2004).

All mice were kept at room temperature of 22 ± 3°C, a relative humidity of 50% ± 20%, and a 12-h light/dark cycle. The mice were housed five per group per sex in shoebox cages with hardwood shavings as bedding. Prior to treatment, mice were quarantined for 1 week to evaluate any signs of disease and weight gain. During the 1-week acclimatization period, the mice got free access to fresh water and basic diet. After quarantine, the mice of each sex were assigned to different groups on the basis of body weights using a randomized block model, ensuring that the body weights by gender of all groups were homogeneous according to the statistical analysis at the beginning of the study.

### Analysis of MEQ in the Test Diet

The basic feed (Mice Maintenance Feed, Ground Fine from Center of Laboratory Animals of Hubei Province, Wuhan, PR China), were produced according to the Chinese standard “Laboratory animal rats and mice feed” (Chinese Standard GB14924.3, 2010). In the carcinogenicity study, the basic feed was used as the control diet and was used to preparation of the test diets by incorporation with MEQ weekly. The basic feed was prepared every 2 weeks and mixed separately by group. MEQ mixtures in the diet (25, 55, and 110 mg/kg) were required to be stable for up to 2 weeks. Stability and homogeneity of MEQ in diet formulations for 2 weeks at room temperature storage were verified by HPLC with the method described by Fang et al. (2006). Ensure that good homogeneous distribution and stability of MEQ was present in the diets.

### Experimental Design

Kun-Ming mice were randomly assigned to four groups (50 mice/group/sex) based on their body weights, and each group of mice were fed the basal diet mixed with 0, 25, 55, and 110 mg/kg MEQ for a total period of 78 weeks (**Table 1**). According to the Organization for Economic Cooperation and Development (OECD) Guideline 453, US FDA Redbook 2000 (FDA, 2000a,b,c) and Procedures for toxicological assessment of food in China, the following points should be taken into account when selecting the appropriate dosage in the design of carcinogenicity study: (a) the highest dose should cause a certain toxic effect but not produce death or severe suffering; (b) the dose should not exceed 5% of the diet for non-nutritive additives; (c) the lowest dose was recommended to be 1–3 times of the clinical dose (Chinese Standard GB15193.17, 2003; OECD, 2009). In a previous sub-chronic and chronic toxicity study, MEQ (110 mg/kg diet) increased the K^+^ level in plasma without growth inhibition (Ihsan, 2011). The medium dosage of 55 mg/kg is within 2–4 times of the minimal dose (25 mg/kg) according to the suggestion regarding dose level spacing in OECD Guideline 453. Therefore, the 110 mg/kg diet was selected as the high dose, and 55 mg/kg for the middle and 25 mg/kg for the low dose, respectively.

**Table 1:** Daily intake in carcinogenicity study of mequindox in KM mice (Mean ± SD).

Group number^a^	Treatment	Concentrations (mg/kg diet)	Dosage^b^ (mg/kg b.w./day)	
			Female	Male
1	Basal diet	Control	0	0
2	Mequindox	M25	2.25 ± 0.26	1.72 ± 0.12
3	Mequindox	M55	4.99 ± 0.67	3.59 ± 0.24
4	Mequindox	M110	10.30 ± 1.12	7.06 ± 0.26

*SD, standard deviation; ^*a*^50 mice/sex/group; ^*b*^Counted from weekly individual feed consumptions and nominal dietary concentration*. *M, mequindox; M25, 25 mg/kg diet; M55, 55 mg/kg diet; M110, 110 mg/kg diet.*

### Parameters Evaluated

#### Clinical Observation and Survival

Mice were individually handled and carefully examined for abnormal behaviors and appearance before treatment. Each mouse was checked for mortality at least twice daily throughout the carcinogenicity study. The detailed physical examination were conducted on the base of behavioral changes, the visible changes in skin, fur, eyes, mucous membranes, secretions and excretions. Changes in the gait were assessed weekly by allowing the animal to walk freely. The onset, location, size and progression of masses were recorded by palpation every 2 weeks. Ophthalmic examinations were conducted for all the mice four intervals during the whole study period.

#### Body Weights and Feed Consumption

Individual body weight data and feed consumption (g/animal/day) data were obtained weekly, beginning the experimental diet administration through 13 weeks. After13 weeks, every 4 weeks for the body weight, and every 3 months for feed consumption were recorded accordingly throughout the carcinogenicity study. Before necropsy the fasted weights for all the mice were recorded. Individual body weight, feed consumption and mean daily intake of MEQ (mg/kg b.w./day) were calculated as described (Wang et al., 2015a,b; Liu et al., 2017a).

#### Clinical Pathology

The number of sacrificed mice was 10 per group (5 mice/sex) at weeks 26 and 52. The remaining survival mice were sacrificed at the final scheduled necropsy (78 weeks). According to the OECD and FDA Redbook guidelines, standard hematology and serum chemistry parameters should be evaluated at weeks 26, 52, and 78. Following an overnight fast, animals were anesthetized and euthanized, and then, the blood samples were collected from each mice for hematology and serum biochemistry. K^+^ EDTA was used as an anticoagulant in blood for the hematology tests. For clinical chemistry, blood samples were placed in serum tubes at room temperature for approximately 30 min to obtain serum aliquots. After clotting, the blood tubes were centrifuged at 3000 rpm for 10 min by using Himac CR 21 G centrifuge (Hitachi, Tokyo, Japan). The supernatants were decanted and stored at -20°C for further serum biochemistry analysis.

##### Hematology

Hematological measurements and calculations were performed by using Coulter HmX *Hematology* Analyzer (Beckman Coulter Inc., Fullerton, CA, United States). The following twelve indicators were evaluated in hematology: RBC, HGB concentration, HCT, MCV, MCH, MCHC, RDW, PCT, blood PLT, MPV, PDW, and WBC.

##### Clinical chemistry

Serum chemistry was assessed using Synchron Clinical System *CX4* (Beckman Coulter, Brea, CA, United States) according to the manufacturer’s directions (Beijing Leadman Biochemistry Technology Co., Ltd., Beijing, China). Parameters in serum chemistry included ALB, ALT, AST, TG, TP, URE, TCHO, CREA, GLU, CL^-^, ALP, UA, TBA and so on.

#### Evaluation of Systemic Genotoxic Damage

The cell suspensions from femur bone marrow was obtained from the mice (*n* = 5 mice/sex/group). Bone marrows were flushed with 1 mL fetal bovine serum and then dropped onto microscope slides. Before microscopic analysis, the cells were fixed with absolute methanol and stained with 5% Giemsa stain for 5 min. For each mouse, 1000 polychromatic erythrocytes (PCE) were counted to determine the micronucleus frequencies and record the micronucleus occurrence rate per one thousand PCE. The proportion of PCE to normochromatic erythrocytes (NCE) was also evaluated.

#### Ophthalmic Examinations

Ocular examinations were performed on all the mice by a board-certified veterinary ophthalmologist prior to randomization, at approximately 26, 52, and 78 weeks.

#### Macroscopic Examination and Relative Weight of Organs

The mice were euthanized and a complete necropsy was conducted for each mice. The macroscopic examination of organs/tissues included the visible lesions on the external surface. In this study, the standard list of tissues recommended by Society of Toxicological Pathology (STP) was examined in the macroscopic examination. Each mice received a gross necropsy and microscopic examination of standard tissues including: brain, spinal cord, eye, sub-maxillary gland, thyroid gland, thymus, heart, aorta, lung, sternum, liver, spleen, pancreas, kidney, adrenal, stomach, mesenteric lymph node, reproductive tract, urinary bladder, femoral nerve, femoral bone marrow, and mammary gland with skin. The organs/tissues were carefully examined macroscopically and gross lesions were recorded. A complete list of organs and tissues (Mettler-Toledo PL 203, United States), such as brain, heart, lungs, kidneys, liver, spleen, adrenal glands, testes (male), and ovary (female) was weighed separately to calculate the organ weights per 100 g body weight (relative organ weight).

#### Microscopic Examination

The microscopic examination was conducted on the gross lesions and the tissues from all the mice that were found dead or at the scheduled necropsies. The tissues from each mice, with the exception of the testes, were preserved in 10% neutral-buffered formalin and slides were prepared for histopathological examination. Testes were placed in Bouin’s fixative. Histopathological examination was conducted by using routine paraffin embedding technique. Sections (5 μm thick) were stained with H&E, and then the morphological alterations were examined under light microscopy.

### Statistical Analysis

Statistical analyses were performed by comparing the treatment groups with the control group using SPSS11.5 program. Values are presented as mean ± standard deviation. Levene’s test was performed to examine variance homogeneity. If the variance was homogeneous, the data was subjected to one-way ANOVA, if not, they were analyzed by the Kruskal–Wallis non-parametric ANOVA. If either of the tests showed a significant difference among the groups, the data was analyzed by the multiple comparison procedure of the Dunnett’s test. ANOVA was used to analyze body weights, feed consumption, organ weight data, relative organ weight, and clinical pathology values. The incidences of histopathological lesions and tumor incidences were evaluated with the Fisher’s exact probability test (Doi et al., 2006; Liu et al., 2017a). The two-sided level of statistical significance was preset at *p* < 0.05 or 0.01.

## Results

### Clinical Signs, Mortality and Survival Observation

**FIGURE 2:**
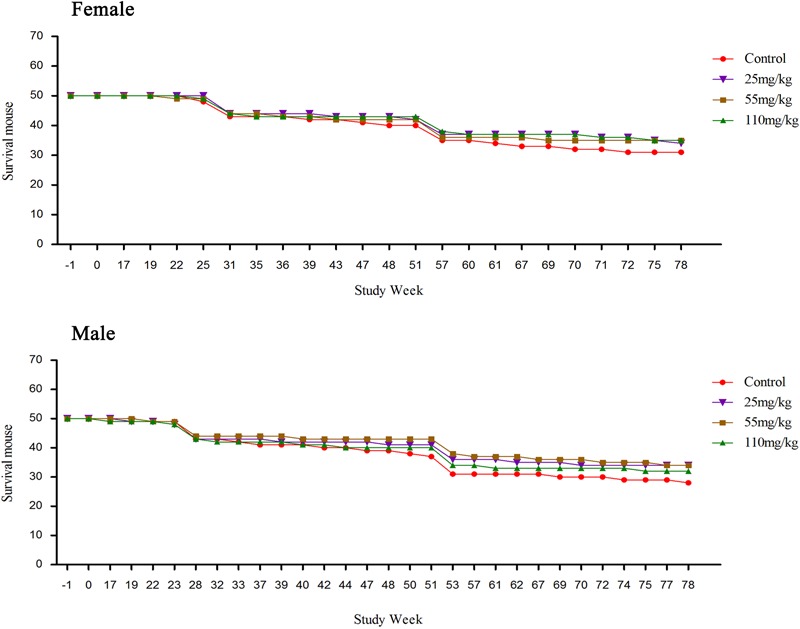
The survival data of female and male mouse in the carcinogenicity study.

Compared to the control, the survival rate was increased in both MEQ treated groups (both sexes). No obvious clinical signs were found in any groups before 16 weeks. The death of the mice was observed from week 17, detailed information was shown in Supplementary Table S1. The numbers of dead mice during the carcinogenicity study were 21, 12, 11, and 13 in the control, M25, M55, and M110, respectively. After the pathological examination, the mice were found died due to hepatitis, pneumonia, rupture of the spleen, uterus suppuration or enteritis (Supplementary Table S1). The survival mouse in the treated groups was higher than that in the control group (**Figure 2**). The numbers of accidental deaths and sacrifice at weeks 26 and 52 in control, 25, 55, and 110 mg/kg group were 19 (9 + 10), 16 (6 + 10), 15 (5 + 10), and 15 (5 + 10) for females, respectively; and 22 (12 + 10), 16 (6 + 10), 16 (6 + 10), and 18 (8 + 10) for males, respectively. Excluding accidental deaths and pre-scheduled necropsy, the percentage of survival at the final scheduled necropsy was 62.0% (31/50), 68.0% (34/50), 70.0% (35/50), and 70.0% (35/50) for control group, the 25, 55, and 110 mg/kg group females, respectively. For males, the percentage of survival at the final scheduled necropsy, excluding accidental deaths and pre-scheduled necropsy, was 56.0% (28/50), 68.0% (34/50), 68.0% (34/50), and 64.0% (32/50) for control group, and the 25, 55, and 110 mg/kg groups, respectively.

In the carcinogenicity study, non-adverse clinical observations of soft feces were found in all the MEQ treated groups. There was no significantly difference between the tested article and control groups in the incidence of palpable masses, and clinical findings in surviving mice. All the clinical findings were limited to single mice, and were common findings for KM mice of similar age.

### Body Weights

**FIGURE 3:**
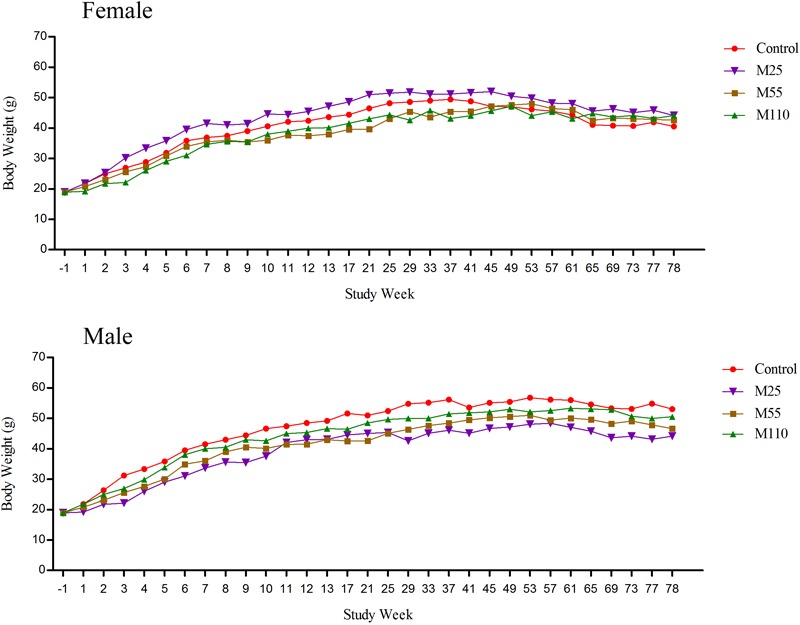
Mean body weights of female and male mice following dietary exposure to MEQ in the carcinogenicity study. (●) Control group; (▼) MEQ group (25 mg/kg); (■) MEQ group (55 mg/kg); (▲) MEQ group (110 mg/kg).

The body weights of female and male mice in the carcinogenicity study were presented in **Figure 3**. The body weights in the M25 mg/kg female group was higher than that in the control group. Body weights in the female mice from the 1st to 8th weeks and 37th to 49th weeks, were controls > M55 mg/kg > M110 mg/kg, while from 9th to 25th weeks, 53th to 61th weeks and 65th to 78th weeks, the body weights were controls > M110 mg/kg > M55 mg/kg, M55 mg/kg > controls > M110 mg/kg, and M110 mg/kg > M55 mg/kg > controls, respectively. Body weights in male mice from the first to 11th weeks and 25th to 78th weeks, were controls > M110 mg/kg > M55 mg/kg > M25 mg/kg; while from 12th to 21 weeks, the body weights were controls > M110 mg/kg > M25 mg/kg > M55 mg/kg.

### Feed Consumption and MEQ Intake

**FIGURE 4:**
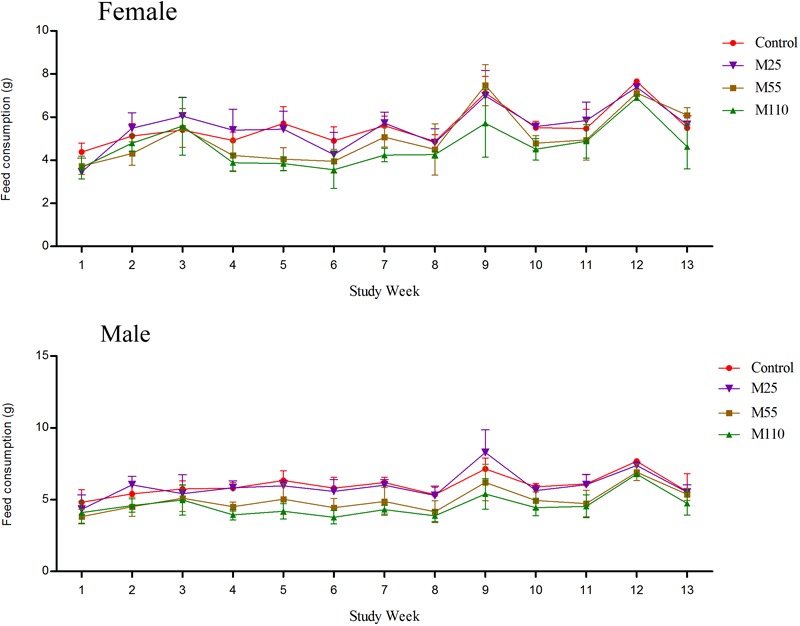
Feed consumption of female and male mice following dietary exposure to MEQ in the carcinogenicity study. (●) Control group; (▼) MEQ group (25 mg/kg); (■) MEQ group (55 mg/kg); (▲) MEQ group (110 mg/kg).

There was a lack of consistency in feed consumption of female and male mice from 1st week to 13th week for the reason that several values were either similar to or higher than the control groups (**Figure 4**). Compared with controls, feed consumption of females increased significantly at the weeks of 2nd to 4th, 7th, 10th, 11th, and 13th weeks at M25 mg/kg group, while it decreased significantly at the first, 5th, 6th, 8th, 9th, and 12th weeks, respectively. Significant increase of feed consumption was noted at the 3rd, 9th, and 13th weeks at M55 mg/kg group, while it decreased significantly at the first, 2nd, 4th to 8th and 10th to 12th weeks, respectively. In M110 mg/kg female group, there was significant increase in feed consumption at 3rd week, while a significant decrease in feed consumption at the first, 2nd, and from 4th to 13th weeks, respectively. For males, there was a significant increase in feed consumption in M25 mg/kg group at the 2nd, 4th, and 9th weeks, and significant decrease at the first, 3rd, from 5th to 8th and 10th to 13th weeks, respectively. Feed consumption of males decreased significantly from 1 to 13th weeks in M55 and M110 mg/kg groups. Compared with controls, the feed consumption in all treated groups decreased significantly throughout the study from the 14th to 78th weeks.

Mequindox intakes per kg body weight per day achieved in the study were calculated based on the feed intakes and body weights as shown in **Table 1**.

### Hematological Examination

Hematology data of weeks 26 and 52, and week 78 were showed in Supplementary Table S2 and **Table 2**, respectively. In females, significant decrease of RDW was noted at the 26th weeks in all the treated groups (Supplementary Table S2); significant increase of RBC and significant decrease of MPV were found at the 26th weeks in M55 and M110 mg/kg groups (Supplementary Table S2); a marked increase of MCV, HGB, and PLT were observed in M25, M55, and M110 mg/kg groups at the 26th weeks, respectively. A significant increased of PDW in M110 mg/kg female group were noted at both 26th and 52th weeks. A significant increase of RBC, HGB, HCT, MCV, MCH, and MCHC was observed at the 52th weeks in M110 mg/kg female group (Supplementary Table S2). A significant changed level of MCHC in M55 mg/kg female group was noted at the 26th and 52th weeks. For female mice at the 78th weeks, significant increased of HGB, MCH, and PCT were observed on both M25 and M55 mg/kg groups; a significant decreased of RBC and increased of MCT were noted in all the treated groups; a significant decreased of MCV and increased of RDW were found in both the M55 and M110 mg/kg groups (**Table 2**).

**Table 2:** Hematology parameters of KM mice fed mequindox at week 78 in carcinogenicity study (Mean ± SD).

	Females				Males			
	Control (*n* = 32)	M25 (*n* = 33)	M55 (*n* = 30)	M110 (*n* = 32)	Control (*n* = 27)	M25 (*n* = 29)	M55 (*n* = 31)	M110 (*n* = 34)
RBC (10^12^/L)	6.0 ± 1.1	4.4 ± 1.5*	5.0 ± 1.5**	3.9 ± 1.2**	5.1 ± 1.1	4.8 ± 1.2	4.4 ± 1.2	4.1 ± 1.1
HGB (g/L)	105.5 ± 17.6	250.7 ± 40.5**	267.3 ± 60.6**	67.7 ± 14.7**	90.6 ± 18.9	242.5 ± 53.4**	220.3 ± 67.1**	62.0 ± 18.5**
HCT (%)	0.27 ± 0.05	0.20 ± 0.06*	0.20 ± 0.08*	0.17 ± 0.06**	0.22 ± 0.05	0.22 ± 0.05	0.23 ± 0.02	0.18 ± 0.05
MCV (fl)	44.6 ± 0.7	43.5 ± 1.5	42.8 ± 2.1**	43.3 ± 2.0*	42.4 ± 1.8	47.7 ± 3.5**	47.7 ± 2.4**	42.8 ± 1.5
MCH (Pg)	17.6 ± 0.8	38.8 ± 12.1**	38.5 ± 13.4**	21.2 ± 7.6	18.4 ± 5.1	51.1 ± 5.8**	49.3 ± 4.6**	16.8 ± 3.9
MCHC (g/L)	394.5 ± 18.3	427.7 ± 38.9*	444.7 ± 104.7	386.9 ± 111.7	431.3 ± 111.4	463.9 ± 39.6	520.8 ± 95.8	367.1 ± 37.3
RDW (%)	17.7 ± 0.9	22.6 ± 9.5	25.7 ± 8.2**	20.1 ± 3.9*	27.5 ± 7.6	18.9 ± 6.0*	16.3 ± 2.7**	21.0 ± 4.7*
MPV (fl)	5.1 ± 0.6	5.1 ± 0.5	5.3 ± 0.7	5.4 ± 0.5	5.1 ± 0.3	4.9 ± 0.7	5.0 ± 0.5	5.4 ± 0.6
PCT (%)	0.23 ± 0.07	0.42 ± 0.06**	0.34 ± 0.09*	0.08 ± 0.04**	0.19 ± 0.06	0.5 ± 0.2**	0.55 ± 0.2**	0.03 ± 0.01**
PDW (%)	15.9 ± 0.3	15.3 ± 1.1	15.7 ± 0.8	15.5 ± 0.9	16.3 ± 0.7	15.4 ± 1.0	15.0 ± 0.4**	15.4 ± 0.6**

*SD, standard deviation. M, mequindox; M25, 25 mg/kg diet; M55, 55 mg/kg diet; M110, 110 mg/kg diet. ^∗^Significantly different from control group at p < 0.05. ^∗∗^Significantly different from control group at p < 0.01*.

In males, there was significant decrease of MPV at the 26th weeks in M55 and M110 mg/kg groups; significant increases of PLT and PDW at the 26th weeks in M110 mg/kg group (Supplementary Table S2). Significant increased of HGB and significant decreased of MCHC were noted in M110 mg/kg male group at the 52th weeks (Supplementary Table S2). Significant increase of HGB, MCV, MCH, and PCT in males was noted at the 78th weeks in M25 and M55 mg/kg groups (**Table 2**). At the 78th weeks in males, there was significant decrease of RDW in all the treated groups; significant decrease of PDW was found in M55 and M110 mg/kg groups; significant decreases of HGB and PCT were noted in M110 mg/kg group (**Table 2**).

### Biochemical Changes

The results of serum clinical chemistry at weeks 26 and 52, and at week 78 are presented in Supplementary Table S3 and **Table 3**, respectively. At the 26th week in female mice, significant decrease of GLU was noted in M25 and M55 mg/kg groups, and significant decrease of CL^-^, and UREA and TBA were observed in M25 and M110 mg/kg groups, respectively (Supplementary Table S3). In females, there was significant decrease in ALP, ALT, LDHD, and TCHO, and significant decrease in ALP, LDHD, Ca^2+^, and TBA, and significant decrease in ALP, Ca^2+^, TCHO, and GGT at the 52th week in M25, M55, and M110 mg/kg group, respectively; a significant increase in GLU was found at the 52th week in M55 and M110 mg/kg groups, while significant increase in AST and TBA was noted at the 52th week in M110 mg/kg group (Supplementary Table S3). At the 78th week in female mice, significant increase of PD and Na^+^, and significant decrease of ALT, BUN, and TG were noted in all the treated groups; significant increase of TP and CL^-^, and significant decrease of AST were found in M25 mg/kg and M55 mg/kg groups; significant decrease of ALP, and significant decrease of CREA and LDHD were noted in M25 and M55 mg/kg group, respectively; significant increase of LDHD, and significant decrease of CREA and GLU were noted in M110 mg/kg group (**Table 3**).

**Table 3:** Serum clinical chemistry parameters of KM mice fed mequindox at week 78 in carcinogenicity study (Mean ± SD).

	Females				Males			
	Control (*n* = 32)	M25 (*n* = 33)	M55 (*n* = 30)	M110 (*n* = 32)	Control (*n* = 27)	M25 (*n* = 29)	M55 (*n* = 31)	M110 (*n* = 34)
ALB (g/L)	37.3 ± 7.7	41.7 ± 6.5	42.9 ± 7.1	43.2 ± 6.9	38.7 ± 7.9	33.0 ± 4.2	31.6 ± 5.7	42.84 ± 2.65
ALP (U/L)	139.6 ± 36.7	82.3 ± 21.6**	148.3 ± 52.7	116.7 ± 35.6	107.6 ± 18.2	62.2 ± 13.1**	43.6 ± 6.9**	78.8 ± 25.4*
ALT (U/L)	156.6 ± 60.5	39.9 ± 10.9**	30.0 ± 8.4**	49.5 ± 19.6**	88.4 ± 25.7	32.2 ± 2.6**	30.1 ± 6.3**	75.3 ± 13.3
AST (U/L)	274.7 ± 54.2	158.2 ± 55.0**	153.3 ± 68.8**	231.9 ± 68.1	252.3 ± 41.6	96.0 ± 18.6**	109.3 ± 40.1**	214.4 ± 48.1
BUN (mmol/L)	15.7 ± 3.4	8.1 ± 1.8**	8.4 ± 1.9**	7.6 ± 0.8**	15.8 ± 3.3	7.9 ± 1.4**	7.5 ± 0.7**	8.6 ± 1.1**
CREA (μmol/L)	25.3 ± 5.8	19.8 ± 5.9	14.6 ± 4.2**	16.7 ± 2.7**	25.3 ± 3.9	13.8 ± 1.7**	12.9 ± 4.3**	15.0 ± 5.0**
LDHD (U/L)	737.3 ± 125.9	610.3 ± 157.6	526.3 ± 119.3**	1714.6 ± 288.9**	656.9 ± 101.9	523.0 ± 60.3*	464.2 ± 115.3**	2028.6 ± 243.4**
TG (mmol/L)	4.9 ± 1.2	2.2 ± 0.6**	2.1 ± 0.6**	2.3 ± 0.8**	3.9 ± 1.1	2.0 ± 0.5**	2.9 ± 0.7	2.8 ± 0.8*
Ca^++^ (mmol/L)	2.7 ± 0.4	2.7 ± 0.3	2.4 ± 0.3	2.5 ± 0.20	3.0 ± 0.5	2.6 ± 0.4	2.5 ± 0.3	2.5 ± 0.2*
Cl^-^ (mmol/L)	123.5 ± 2.7	130.7 ± 9.5*	137.2 ± 14.0**	121.7 ± 8.1	124.1 ± 3.6	130.7 ± 10.7	125.1 ± 4.0	123.9 ± 7.8
Na^+^ (mmol/L)	95.2 ± 11.8	159.9 ± 16.8**	147.5 ± 13.8**	128.7 ± 3.2**	96.9 ± 10.4	168.7 ± 8.7**	160.8 ± 8.7**	129.6 ± 4.9**
PD (mmol/L)	3.3 ± 0.3	5.7 ± 1.2*	4.1 ± 0.8*	4.7 ± 0.6**	3.4 ± 0.3	5.6 ± 0.3**	4.4 ± 0.8*	5.3 ± 1.4**
TBA (μmol/L)	31.6 ± 7.1	35.1 ± 7.1	29.4 ± 9.5	28.9 ± 7.9	28.9 ± 8.8	12.8 ± 3.8**	25.9 ± 3.2	68.0 ± 9.6**
GLU (mmol/L)	2.7 ± 0.9	2.4 ± 0.8	2.9 ± 0.9	1.2 ± 0.6**	3.6 ± 1.1	4.6 ± 0.7	3.6 ± 1.1	1.8 ± 0.3**
TP (g/L)	77.0 ± 16.7	97.6 ± 22.5*	111.6 ± 26.1**	92.5 ± 17.5	79.0 ± 15.9	70.8 ± 5.2	71.9 ± 17.0	87.8 ± 15.4

*SD, standard deviation. M, mequindox; M25, 25 mg/kg diet; M55, 55 mg/kg diet; M110, 110 mg/kg diet. ^∗^Significantly different from control group at p < 0.05. ^∗∗^Significantly different from control group at p < 0.01.*

In males, there was significant decrease in ALB at the 26th week in all the MEQ treated groups; significant decrease of ALP and GLU were noted at the 26th week in M25 mg/kg group; significant decrease of CREA and significant increase of ALT were noted at the 26th week in M55 mg/kg group (Supplementary Table S3). At the 52th week in male mice, significant increase of ALT and significant decrease of TCHO and GGT were observed in all treated groups; significant increase of TG and TP were noted in M25 and M55 mg/kg groups; significant decrease of CREA and TBA were found in M55 and M110 mg/kg groups; significant increase of UA was noted in M25 and M110 mg/kg groups; significant decrease of LDHD, significant increase of ALB, and significant decrease of ALP and AST were observed in M25, M55, and M110 mg/kg groups, respectively (Supplementary Table S3). Significant decrease of ALP, BUN, and CREA, and significant increase of PD and Na^+^ in males was noted at the 78th weeks in all treated groups (**Table 3**). At the 78th weeks in males, there were significant decreases in ALT, AST, and LDHD in M25 and M55 mg/kg groups; significant decrease of TG was found in M25 and M110 mg/kg groups; significant decreases of TBA was found in M25 mg/kg group; significant increases of LDHD and TBA, and significant decreases of Ca^2+^ and GLU were observed in M110 mg/kg group (**Table 3**).

### Ophthalmic Examinations

There were no ophthalmic lesions observed in any MEQ treated groups which show indication of toxicity (data not shown). All the findings found throughout the study were marked as typical in prevalence and appearance for mice of this age, and were not related to administration of the test article of MEQ.

### Organ Weight, Relative Organ Weights and Macroscopic Examination

#### Organ Weight

In the females, significant decrease of ovary and significant increase of kidney were noted in M25 mg/kg group at the 26th and 52th week, respectively (Supplementary Table S4). Significant decrease of spleen, and significant decrease of heart and liver were observed males at the 26th week in M25 and M55 groups, respectively (Supplementary Table S4). At the 78th week, there was significant increase of spleen in females in M25 and M55 mg/kg groups; significant increase of lungs and kidney, significant decrease of liver and brain were found in females in M25 and M110 mg/kg group, respectively (**Table 4**). Significant decrease of spleen in M55 and M110 mg/kg groups and significant decrease of brain and testis in M110 mg/kg group were found at the 78th week in males (**Table 4**).

**Table 4:** Organ weights (g) in KM mice fed mequindox at week 78 in carcinogenicity study (Mean ± SD).

	Females				Males			
	Control (*n* = 31)	M25 (*n* = 34)	M55 (*n* = 35)	M110 (*n* = 35)	Control (*n* = 28)	M25 (*n* = 34)	M55 (*n* = 34)	M110 (*n* = 32)
Final body weight (g)	40.5 ± 6.7	43.9 ± 7.1*	47.0 ± 7.1	46.2 ± 6.9	51.8 ± 4.9	43.2 ± 3.3*	47.3 ± 3.5	46.6 ± 1.5
Heart	0.2 ± 0.03	0.2 ± 0.06	0.2 ± 0.04	0.2 ± 0.03	0.2 ± 0.02	0.2 ± 0.03	0.3 ± 0.07	0.2 ± 0.07
Liver	2.2 ± 0.3	2.0 ± 0.3	2.2 ± 0.4	1.7 ± 0.3**	2.2 ± 0.3	2.0 ± 0.3	2.0 ± 0.27	1.8 ± 0.3
Spleen	0.09 ± 0.03	0.1 ± 0.02*	0.2 ± 0.05**	0.09 ± 0.03	0.1 ± 0.02	0.2 ± 0.04	0.09 ± 0.02*	0.06 ± 0.02**
Lungs	0.2 ± 0.08	0.3 ± 0.06*	0.3 ± 0.06	0.3 ± 0.06	0.3 ± 0.04	0.3 ± 0.03	0.3 ± 0.04	0.2 ± 0.04
Kidney	0.5 ± 0.1	0.6 ± 0.08*	0.6 ± 0.07	0.5 ± 0.04	0.7 ± 0.05	0.7 ± 0.08	0.7 ± 0.12	0.7 ± 0.1
Adrenal	0.02 ± 0.006	0.02 ± 0.004	0.03 ± 0.002*	0.03 ± 0.003*	0.01 ± 0.006	0.02 ± 0.005*	0.02 ± 0.006*	0.02 ± 0.005*
Brain	0.5 ± 0.04	0.5 ± 0.02	0.5 ± 0.03	0.4 ± 0.03*	0.5 ± 0.03	0.5 ± 0.03	0.4 ± 0.02	0.4 ± 0.02**
Ovary and Uterus	0.2 ± 0.1	0.8 ± 1.1	0.2 ± 0.07	0.2 ± 0.06	-	-	-	-
Testis	-	-	-	-	0.3 ± 0.05	0.2 ± 0.04	0.2 ± 0.05	0.2 ± 0.05*

*SD, standard deviation; bw, body weight. M, mequindox; M25, 25 mg/kg diet; M55, 55 mg/kg diet; M110, 110 mg/kg diet*. *^∗^Significantly different from control group at p < 0.05; ^∗∗^Significantly different from control group at p < 0.01.*

#### Relative Organ Weights

In females, significant increase of relative organ weights of uterus and heart were observed in M55 mg/kg group at 26th and 52th week, respectively; significant decrease of relative organ weights of livers were found in all MEQ treated groups at 78th week; significant decrease of relative organ weights of uterus and brains were noted in M110 mg/kg group at 52th and 78th week, respectively; significant increase of relative organ weights of lungs and ovary and uterus, and significant decrease of relative organ weights of brains were observed in M25 and M55 mg/kg groups at 78th week (Supplementary Table S5 and **Table 5**).

**Table 5:** Relative organ weights (mg/g final bw) in KM mice fed mequindox at week 78 in carcinogenicity study (Mean ± SD).

	Females				Males			
	Control (*n* = 31)	M25 (*n* = 34)	M55 (*n* = 35)	M110 (*n* = 35)	Control (*n* = 28)	M25 (*n* = 34)	M55 (*n* = 34)	M110 (*n* = 32)
Final body weight (g)	40.5 ± 6.7	43.9 ± 7.1*	47.0 ± 7.1	46.2 ± 6.9	51.8 ± 4.9	43.2 ± 3.3*	47.3 ± 3.5	46.6 ± 1.5
Heart	4.0 ± 0.5	4.2 ± 1.6	4.2 ± 0.9	4.1 ± 0.9	4.7 ± 0.6	5.3 ± 0.8	5.5 ± 1.6	4.6 ± 0.8
Liver	54.4 ± 4.1	45.2 ± 5.9**	46.6 ± 5.5**	37.8 ± 9.4**	43.5 ± 6.7	45.1 ± 4.1	41.3 ± 3.5	41.6 ± 7.1
Spleen	2.1 ± 1.2	2.5 ± 0.8	2.1 ± 0.7	1.7 ± 0.7	2.6 ± 0.4	2.7 ± 0.9	2.3 ± 0.8	1.4 ± 0.4**
Lungs	6.1 ± 1.1	7.6 ± 1.4*	6.3 ± 1.5	6.5 ± 1.2	5.3 ± 1.1	6.3 ± 0.7	6.8 ± 1.0	6.0 ± 1.7
Kidney	11.5 ± 1.4	13.3 ± 2.7	11.8 ± 1.2	11.7 ± 1.8	14.3 ± 1.2	16.6 ± 1.8	14.7 ± 2.1	14.5 ± 2.7
Adrenal^a^	0.2 ± 0.08	0.3 ± 0.01*	0.3 ± 0.03*	0.4 ± 0.06**	0.2 ± 0.1	0.3 ± 0.08*	0.3 ± 0.01*	0.3 ± 0.07*
Brain	11.7 ± 1.5	11.6 ± 2.3	10.0 ± 2.1*	9.5 ± 1.8*	8.9 ± 1.4	10.6 ± 0.9	9.4 ± 0.7	8.4 ± 1.8
Ovary and Uterus	3.2 ± 1.0	6.9 ± 1.8**	3.7 ± 1.2	4.1 ± 1.3	-	-	-	-
Testis	-	-	-	-	5.1 ± 0.8	4.6 ± 0.8	4.2 ± 0.7	4.5 ± 1.2

*SD, standard deviation; bw, body weight. M, mequindox; M25, 25 mg/kg diet; M55, 55 mg/kg diet; M110, 110 mg/kg diet. ^*a*^The unit of relative organ weights of Adrenal is g/10g. ^∗^Significantly different from control group at p < 0.05; ^∗∗^Significantly different from control group at p< 0.01.*

In males, there were significant increase of relative organ weights of brains and testis at all treated groups at 26th week, while significant increase of relative organ weights of livers were observed in all treated groups at 52th week (Supplementary Table S5). In males at 52th week, there were significant increase of relative organ weights of kidney, testis, and brains in M25, M55, and M110 mg/kg groups, respectively (Supplementary Table S5). A significant increase of relative organ weights of spleen was noted in M25 mg/kg group at 26th week, and in M110 mg/kg group at 78th week, respectively (Supplementary Table S5 and **Table 5**).

#### Macroscopic Examination

The gross macroscopic findings that were noted at the scheduled necropsy indicated the obvious toxicity in liver, kidney, testis, uterus, and ovary that were considered associated with the administration of MEQ. Macroscopic observations included rough surface of the kidneys and swelling of the spleen in MEQ treated groups; higher incidence of white areas in the liver and mammary gland masses in the M55 and M110 mg/kg groups; higher incidence of skin masses, uterus cysts oophoritic cyst in the M25, M55, and M110 mg/kg female groups; and higher incidence of swelling of testis and epididymal cyst in the M25, M55, and M110 mg/kg male groups. Reduced body weight gain was considered to play a role in the incidence of gross necropsy findings. However, the reduced body weight gain was not observed in these mouse. Thus, these gross necropsy observations were considered directly related to the administration of the test article of MEQ.

### Systemic Genotoxic Damage

The results for micronucleated polychromatic erythrocytes (MN-PCE) in mice (*n* = 5 mice/sex/group) at the 78th week are presented in **Table 6**. Compared with control group, a significant increase in the ratio of MN-PCE was found when MEQ were administered at the tested concentrations. Additionally, the ratios of PCE/NCE in each group ranged from 0.6 to 1.2. The results indicated that MEQ was genotoxic for mice *in vivo*.

**Table 6:** Micronucleated polychromatic erythrocytes (MN-PCEs) in mice bone marrow (*n* = 10).

Groups	PCE	MN-PCE	MN-PCE/PCE (ddd)	PCE/NCE
M0	10000	20	2.0 ± 0.9	1.19 ± 0.07
M25	10000	49	4.9 ± 1.5^∗^	1.14 ± 0.08
M55	10000	83	8.3 ± 1.6^∗∗^	1.15 ± 0.06
M110	10000	94	9.4 ± 2.9^∗∗^	1.16 ± 0.08

*M, mequindox; M25, 25 mg/kg diet; M55, 55 mg/kg diet; M110, 110 mg/kg diet; NCE, normochromatic erythrocytes; PCE, polychromatic erythrocytes. ^∗^Significantly different from negative control value (p < 0.05). ^∗∗^Significantly different from negative control value (p < 0.01).*

### Histopathological Examination

#### Non-neoplastic Findings

At the 26th week, no obvious pathological change was found in M25 mg/kg and control groups, while mild edema and fatty degeneration of liver cells occurred in some mouse in the M55 and M110 mg/kg groups.

In the 52th week, one female mouse with color-deepened uterus, one male mouse with color-deepened edge of liver was noted in the control group. Two mice with enlargement and color-deepened edge of the spleen, one mouse with one side of the vas deferens shrink and yellow, three mice with swelling of one side of the adrenal gland were noted in the M25 mg/kg group. In the M55 mg/kg group, two mice with white lung, one mouse with abnormal enlargement of the kidney, three mice with color-deepened and black spot in the liver were found. One case of the obvious enlargement in uterine, two mice with bilateral renal enlargement, two mice with white lung, one mouse with color-deepened edge of liver, and two mouse with adrenal gland enlargement were observed in the M110 mg/kg group. An obvious testicular lesions such as swelling and brown spot in the surface occurred in all treated groups, and of which the cases were 2, 3, 3 in the M25, M55, and M110 mg/kg groups, respectively.

In the 78th week, the spleen and adrenal gland of two female mice were found significantly larger in control group. Other observation showed enlarged and blackened surface of uterus in eight female mouse; nine mouse with pulmonary hematoma and eight mouse with the enlargement of one side of the adrenal gland in the M25 mg/kg group. In the M55 mg/kg group, five mouse with abnormal uterine, six mouse with one side of the ovary turns black, nine mouse with blacked in the edge of livers, five and eight mouse with enlargement of adrenal gland and spleen, respectively. In M110 mg/kg group, it was found that the cases of the swelling of liver, kidney, adrenal gland and spleen were 11, 6, 7, 4, respectively. Additionally, the obvious lesions in the testicular were found in all MEQ treated groups, and the number were 11, 13, 18 in the M25, M55, and M110 mg/kg groups, respectively.

**FIGURE 5:**
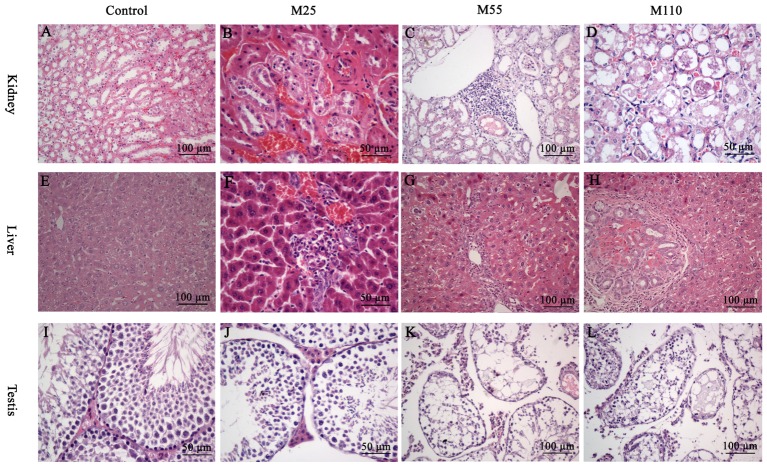
Selected microphotographs of kidney, liver and testis following dietary exposure to MEQ in the carcinogenicity tests (200×, 400×). **(A)** Kidney from control group (200×); **(B)** Kidney from the M25 mg/kg group showing kidney interstitial small blood vessels congestion, glomerular congestion (400×); **(C)** Kidney from the M55 mg/kg group showing aggregation of lymphocyte into a group around the central veins (200×); **(D)** Kidney from the M110 mg/kg group showing degeneration and necrosis of renal tubular epithelial cells (400×); **(E)** Liver from control group (200×); **(F)** Liver from the M25 mg/kg group showing degeneration and necrosis of hepatic cells (400×); **(G)** Liver from the M55 showing neutrophilic infiltrate within and around bile duct (200×); **(H)** Liver from the M110 mg/kg group showing proliferation in bile duct epithelium (200×); **(I)** Testis from the control group (400×); **(J)** Testis from the M25 mg/kg group showing a broadened testicular interstitium (400×); **(K)** Testis at the M55 mg/kg group showing an irregular arrangement as well as a decreased number of spermatogenic cells (200×); **(L)** Testis at the M110 mg/kg group showing necrosis of spermatogonia and spermatocytes in the lumen (200×).

At the 26th, 52th, and 78th, significant histopathological changes in kidney, liver and testis were not observed in control group (**Figures 5A,E,I**). At the scheduled necropsy, expansion of the glomerular capillaries, aggregation of lymphocyte into a group around the central veins, degeneration and necrosis of renal tubular epithelial cells, and the appearance of PRO casts in the tubular lumen were found in kidney in all the treated groups at the 78th week (**Figures 5B–D**); the degeneration and necrosis of hepatic cells under the membrane, neutrophilic infiltrate within and around bile duct, and proliferation in most bile duct epithelium were observed in liver of mouse in all treated groups at the 52th and 78th week (**Figures 5F–H**); compared with the control group, a broadened testicular interstitium, an irregular arrangement and decreased number of spermatogenic cells in the lumen, as well as necrosis of spermatogonia and spermatocytes in the lumen were noted at the 52th and 78th week (**Figures 5J–L**).

#### Neoplastic Findings

**FIGURE 6:**
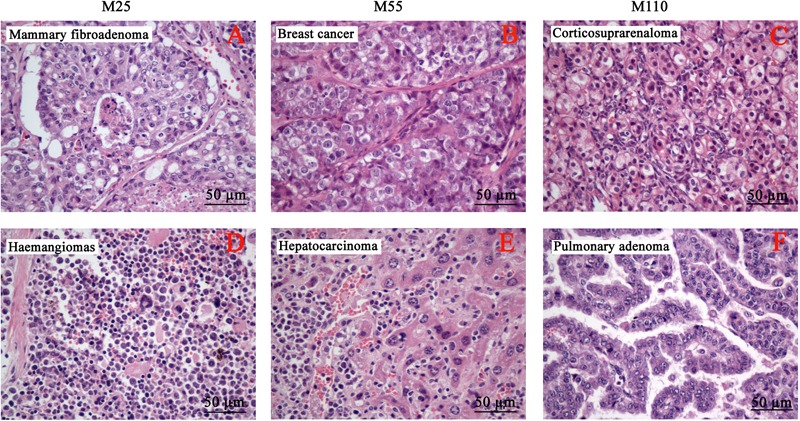
Selected microphotographs of the identified neoplasm in the carcinogenicity study (400×). **(A)** Mammary fibroadenoma in M25 mg/kg group. **(B)** Breast cancer in M55 mg/kg group. **(C)** Corticosuprarenaloma in M110 mg/kg group. **(D)** Haemangiomas in M25 mg/kg group. **(E)** Hepatocarcinoma M55 mg/kg group. **(F)** Pulmonary adenoma M110 mg/kg group.

The neoplastic findings and neoplasm incidence were presented in **Table 7**. The first tumor case appeared in the female at M25 mg/kg group in the 49th week. In the carcinogenicity study, the number of the total survival mice was 343 including 168 males and 175 females. The number of the survival at the final scheduled necropsy were 59, 68, 69, and 67 in control, 25, 55, and 110 mg/kg groups, respectively. Mouse tumors were chiefly six types, including mammary fibroadenoma (**Figure 6A**), breast cancer (**Figure 6B**), corticosuprarenaloma (**Figure 6C**), haemangiomas (**Figure 6D**), hepatocarcinoma (**Figure 6E**), and pulmonary adenoma (**Figure 6F**). For mammary fibroadenoma, the incidence was 7.3% (3/41), 27.3% (12/44), 26.7% (12/45), and 22.2% (10/45) for the control, M25, M55, and M110 mg/kg groups, respectively. The incidence of corticosuprarenaloma was 0% (0/79), 30.7% (27/88), 19.1% (17/89), and 17.2% (15/87) for the control, M25, M55, and M110 mg/kg groups, respectively. The hepatocarcinoma incidence in females was 0% (0/41), 31.8% (14/44), 24.4% (11/45), and 22.2% (10/45) for the control, M25, M55, and M110 mg/kg groups, respectively. In males, the hepatocarcinoma incidence was 2.6% (1/38), 27.3% (12/44), 20.5% (9/44), and 21.4% (9/42) for the control, M25, M55, and M110 mg/kg groups, respectively. The haemangiomas incidence in females was 2.4% (1/41), 27.3% (12/44), 20.0% (9/45), and 15.6% (7/45) for the control, M25, M55, and M110 mg/kg groups, respectively. In males, the hepatocarcinoma incidence was 0 (0/38), 22.7% (10/44), 20.5% (9/44), and 21.4% (9/42) for the control, M25, M55, and M110 mg/kg groups, respectively. The pulmonary adenoma incidence in females was 4.9% (2/41), 27.3% (12/44), 24.4% (11/45), and 26.7% (12/45) for the control, M25, M55, and M110 mg/kg groups, respectively. In males, the pulmonary adenoma incidence was 7.9% (3/38), 25.0% (11/44), 20.5% (9/44), and 21.4% (9/42) for the control, M25, M55, and M110 mg/kg groups, respectively. For breast cancer, the incidence was 2.4% (1/41), 34.1% (15/44), 26.7% (12/45), and 28.9% (13/45) for the control, M25, M55, and M110 mg/kg groups, respectively. These results demonstrated that MEQ was carcinogenic to KM mice.

**Table 7:** Summary of neoplastic findings and neoplasm incidence in carcinogenicity study of mequindox in KM mice.

Females	Control (*n* = 41)	M25 (*n* = 44)	M55 (*n* = 45)	M110 (*n* = 45)
Liver				
Hepatocarcinoma	0 (0%)	14 (31.8%)	11 (24.4%)	10 (22.2%)
Spleen				
Splenic hemangioma	0 (0%)	9 (20.5%)	6 (13.3%)	5 (11.1%)
Artery				
Vascular cancer	1 (2.4%)	3 (6.8%)	3 (6.7%)	2 (4.4%)
Lung				
Adenocarcinoma	2 (4.9%)	12 (27.3%)	11 (24.4%)	12 (26.7%)
Uterus				
Uterine leiomyoma	2 (4.9%)	3 (6.8%)	1 (2.2%)	2 (4.4%)
Intestinal				
Colon cancer	1 (2.4%)	1 (2.3%)	0 (0%)	2 (4.4%)
Subcutaneous				
Breast fibroadenoma,	3 (7.3%)	12 (27.3%)	12 (26.7%)	10 (22.2%)
Breast tumor	1 (2.4%)	15 (34.1%)	12 (26.7%)	13 (28.9%)
Thyroid carcinoma	0 (0%)	2 (4.5%)	0 (0%)	1 (2.2%)
Adrenal gland				
Corticosuprarenaloma	0 (0%)	12 (27.3%)	10 (22.2%)	9 (20.0%)

**Males**	**Control (*n* = 38)**	**M25 (*n* = 44)**	**M55 (*n* = 44)**	**M110 (*n* = 42)**

Liver				
Hepatocarcinoma	1 (2.6%)	12 (27.3%)	9 (20.5%)	9 (21.4%)
Spleen				
Splenic hemangioma	0 (0%)	4 (9.1%)	2 (4.5%)	6 (14.3%)
Lung				
Adenocarcinoma	3 (7.9%)	11 (25.0%)	9 (20.5%)	9 (21.4%)
Intestinal				
Colon cancer	2 (5.3%)	1 (2.3%)	2 (4.5%)	1 (2.4%)
Artery				
Vascular cancer	0 (0%)	10 (22.7%)	9 (20.5%)	9 (21.4%)
Thyroid carcinoma	1 (2.6%)	3 (6.8%)	2 (4.5%)	0 (0%)
Adrenal gland				
Corticosuprarenaloma	0 (0%)	15 (34.1%)	7 (15.9%)	6 (14.3%)

*M, mequindox; M25, 25 mg/kg diet; M55, 55 mg/kg diet; M110, 110 mg/kg diet.*

## Discussion

Mequindox, a relative new compound in QdNOs, is widely applied in livestock owing to its strong inhibitory activities against microbes in China (Liu et al., 2012; Wu et al., 2012; Li et al., 2014; Wang et al., 2016b). Early findings have demonstrated that adrenal toxicity (Huang et al., 2009), testicular toxicity (Ihsan et al., 2011; Liu et al., 2017c,b), kidney and liver toxicity (Huang et al., 2010a; Liu et al., 2017d) induced by MEQ *in vivo*. CBX (World Health Organization [WHO], 1991a) and OLA (World Health Organization [WHO], 1991b), structural analogs of MEQ, were prohibited in food-producing animals due to its potential mutagenic and carcinogenic effects (Wu et al., 2007; Liu et al., 2016). However, as part of the non-clinical safety package, the carcinogenicity study of MEQ has not been performed yet. In the present study, the lifetime carcinogenicity study of MEQ in KM mice was conducted for 18 months. The dietary route of administration was selected to ensure a long period of systemic exposure of MEQ to mice. This study illustrates that MEQ induces adverse effects on hematology, serum chemistry and macroscopic observations. Furthermore, MEQ increased the frequency of micronucleated normochromatic erythrocytes, and caused histopathological and neoplastic changes at the scheduled necropsy, suggesting that MEQ was genotoxic and carcinogenic in KM mice.

Compare to the control, the body weights of mice in the MEQ treated groups were reduced in both of the genders except in the M25 mg/kg female group in most of the dosing period (**Figure 3**). Additionally, significantly reduced in the total amount of feed consumption were noted in the M55 and M110 mg/kg groups, suggesting that M55 and M110 mg/kg diets might have some direct toxicity to KM mice. The weakness and emaciation may attribute to the rejection of the diet and bitter taste or toxic effects of MEQ (Mejia et al., 2009; Ihsan et al., 2010). These results were consistent with those reported for OLA at 60 mg/kg b.w. and CYA at 2500 mg/kg diet (World Health Organization [WHO], 1991b; Fang et al., 2006; Liu et al., 2017a). In hematopoietic system, CYA and QCT showed no hematological toxicity in sub-chronic study in Wistar rats (Fang et al., 2006; Ihsan et al., 2010; Wang et al., 2011b). Here, significant changes in hematological parameters, such as HGB, HCT, MCV, MCH, RDW, PCT, and PDW, were observed in MEQ treated groups, indicating that MEQ had an adverse effect on the hematopoietic system (reduced erythrocyte count, HCT, HGB, and platelets). However, the hematological toxicity was not observed in Wistar rats after sub-chronic exposure to MEQ (Ihsan et al., 2010). Therefore, it was suspected that the different toxic effect of MEQ on the hematopoietic system might be due to the species specificity in the metabolism of MEQ. Our data suggested that the hematological system might be the target of MEQ in mice.

Liver was identified as one of the main target organ for CYA (Wang et al., 2011b), QCT (Wang et al., 2010), and MEQ (Ihsan et al., 2010). It was found that disorganized hepatic cord pattern and cellular swelling, centrilobular liver cell necrosis were caused by MEQ in rats (Ihsan et al., 2010) and mice (Liu et al., 2017d). The liver function markers including ALB, ALT, ALP and AST, can be observed in both cytoplasm and mitochondria of hepatocytes. As known, AST catalyzes the conversion of glutamate and oxaloacetate to aspartate and α-ketoglutarate, while ALT catalyzes the conversion of alanine to pyruvate and glutamate (Ihsan et al., 2010; Wang et al., 2011b). In the present study, the significant decreases of ALP, ALB, ALT, and AST, and the significant decreased relative liver weights were observed in week 52 in MEQ treated male groups and at week 78 in MEQ treated female groups, indicating that MEQ might be harmful to the liver. The histopathological examinations indicated the degeneration and necrosis of hepatic cells under the membrane, neutrophilic infiltrate within and around bile duct, and proliferation in most bile duct epithelium in MEQ treated groups at the 52th and 78th weeks. Taken together, these results confirmed the earlier finding that the liver was a toxic target of MEQ *in vivo* (Ihsan et al., 2010; Liu et al., 2017d). Usually, the values of ALT, AST, ALB, and ALP would increase accompanying the liver disease. However, it was interesting that the values of these indicators were significantly decreased. This result was similar to the previous studies of QCT (Wang et al., 2010) and CYA (Wang et al., 2011b; Liu et al., 2017a) in rats. Thus, it was presumed that the production of ALT and AST was inhibited after chronic exposure of mice to MEQ. The further study should be conducted to illustrate the toxicological significance and mechanisms of the decrease in ALT and AST caused by MEQ *in vivo*.

In the previous studies, adrenal toxicity was observed in the following delivery of MEQ in rats (Huang et al., 2009, 2010a; Ihsan et al., 2010). MEQ was reported to reduce the output of adrenal aldosterone (Huang et al., 2010b), and cause the damage of the adrenal with the decrease of the aldosterone concentration in plasma (Huang et al., 2010a). The concentrations of Na^+^, K^+^, UA, and BUN in the serum are controlled by the kidney and adrenal glands (Huang et al., 2010a,b). In the present study, the significantly changed levels of Na^+^, K^+^, UA, and BUN were noted in MEQ treated groups, indicating that chronic injury in kidney and adrenal glands. This observation was consistent with the histopathological changes in the kidneys and adrenal glands. The earlier findings revealed that MEQ resulted in obvious histological changes in adrenal gland (Huang et al., 2009), liver (Wang et al., 2011c; Liu et al., 2017a) and kidney (Wang et al., 2011c). MEQ and its primary metabolites, *N*1-MEQ and B-MEQ, exhibited adrenal toxicity in H295R cells that originated from a human adrenocortical carcinoma (Wang et al., 2016a). In this study, the histopathological examination revealed the serious lesion in the testis of mice, including a broadened testicular interstitium, an irregular arrangement and decreased number of spermatogenic cells in the lumen, as well as necrosis of spermatogonia and spermatocytes in the lumen. Additionally, the histopathological alterations of uterus and ovary were also found in MEQ treated groups. These results indicated that MEQ had adverse effect on developmental and reproductive system. Therefore, our data indicated that the liver, kidneys, adrenal glands and developmental and reproductive system were the main target organs for toxicity mediated by the MEQ *in vivo*.

It was reported that OLA and CBX had genotoxicity and carcinogenicity (Ihsan et al., 2013b,c; Zhao et al., 2014). OLA (360 mg/kg diet) could result in pulmonary adenoma, adrenal cortical adenoma in males and pulmonary adenoma and ovarian granulosa cell tumors in females (Steinhoff and Gunselmann, 1982, Unpublished). Benign fibromas of the skin was observed in Wistar rats after exposure to OLA at 400 mg/kg diet for 78 weeks (Wang et al., 2011a). Administration of OLA to mice at 0, 40, 120, or 360 ppm by dietary admixture increased the incidence of pulmonary adenoma in both sex (JECFA, 2003). When the rats were fed to OLA at 0, 40, 120, or 360 ppm for up to 104 weeks, only mammary fibroadenoma was significantly increased in OLA treated groups (JECFA, 2003). The chronic toxicity and potential carcinogenicity of CBX were evaluated in rats (Stebbins and Coleman, 1967, Unpublished). CBX was administrated via i.p. and feed, the results suggested that CBX treated groups had a largest number of spontaneous and rare tumors, while a higher incidence of hepatic tumor was observed on rats that received CBX via i.p. (Sykora and Vortel, 1986). In the present study, the significant of tumor incidence was noted in the MEQ treated groups when compared to the control group, indicating the strong carcinogenic effect of MEQ to mice. The mouse tumors were chiefly six types, including mammary fibroadenoma, breast cancer, corticosuprarenaloma, haemangiomas, hepatocarcinoma, and pulmonary adenoma.

It was interesting that the tumor incidence didn’t show dose-dependence, and the higher tumor incidence was noted in the M25 mg/kg group. This result was consistent with the previous findings in the carcinogenicity study of CBX in rats, which suggested that the lower tumor incidence was noted in the high dose group (Stebbins and Coleman, 1967, Unpublished). Therefore, there is a lack of correlation between cancer incidence and administration dose of CBX and MEQ. The formation of the tumor in mice is a multi-step phenomenon. After a mutation is induced by MEQ in mice, additional replication cycles and changes are needed before the mutated cell becomes a tumor cell. Furthermore, the mutated cell must survive within the body and replicate to form a tumor (Zeiger, 2001). Based on this, every step of the formation of the tumor requires the continuous overcoming of the toxic effects of the MEQ. In the present study, the higher toxicity was found in M55 and M110 mg/kg groups, and the toxic effects on the liver, kidney, and testis, were associated with oxidative stress and apoptosis evident by TUNEL assay (data not show). Thus, the growth of tumor cells might be disturbed in the M55 and M110 mg/kg groups. These findings suggested a potential relationship among the dose, general toxicity and carcinogenicity *in vivo*, and further study is required to reveal this relationship.

As reported, B-CBX yield positive response in cell transformation test on BALB/C Swiss 3T3 and chromosomal damage on bone marrow of rat (Pfizer, 1975, Unpublished; Holmes, 1976, Unpublished). B-CBX increased the incidence of tumors, including hepatic tumors, subcutaneous fibromas, haemangiomas and mammary tumor in both sexes of rats (Reinert, 1976, Unpublished). Both CBX and B-CBX was concluded by JECFA as carcinogens that acted by a genotoxic mechanisms (JECFA, 2003). MEQ caused chromosomal aberrations in V79 cells and invoked micronucleus formation in mice (Fang et al., 2006; Huang et al., 2010b; Wang et al., 2011a,c; Ihsan et al., 2013a). Additionally, a recent study revealed that B-MEQ and *N*1-MEQ gave positive results in mouse lymphoma assay (MLA), Ames test, chromosomal aberration assay and bone marrow erythrocyte micronucleus assay (Liu et al., 2016). Here, MEQ significantly increased the frequency of micronucleated normochromatic erythrocytes in the bone marrow cells. Therefore, the MEQ was a genotoxic carcinogen with strong tumorigenic effect in KM mice. In a 2-year dietary carcinogenicity study in SD rats, CYA was found no carcinogenicity (Liu et al., 2017a). CBX and its metabolite, B-CBX, were reported to be tumorigenic, whereas another two primary metabolites of CBX, quinoxaline-2-carboxylic acid (QCA) and methyl carbazate had no carcinogenicity (JECFA, 2003). Considering that the same quinoxaline ring is present in all QdNOs, it was assumed that the side chains might play a critical role in the toxicity of QdNOs. The side chains related metabolites of QdNOs were firmly linked to their genotoxicity (Liu et al., 2016) and oxidative stress (Wang et al., 2015c). Thus, the side chain of QdNOs might be responsible for carcinogenicity. The roles of the side chain and the metabolites of QdNOs in the carcinogenicity need further investigation.

## Conclusion

An assessment of carcinogenicity potential of MEQ was carefully evaluated in KM mice using a dose level of 25, 55, and 110 mg/kg MEQ diet. MEQ was carcinogenicity when administered to KM mice for one and a half years. It increased the incidence of tumors, including mammary fibroadenoma, breast cancer, corticosuprarenaloma, haemangiomas, hepatocarcinoma, and pulmonary adenoma. The present study illustrated that the hematological system, liver, kidneys, and adrenal glands, as well as the developmental and reproductive system were the main targets for MEQ after chronic administration to KM mice. Interestingly, the higher incidence of tumors was noted at the lowest dose (M25 mg/kg) with lower organ toxicity, indicating the potential relationship among the dose, general toxicity and carcinogenicity *in vivo*. Importantly, this study identified MEQ as genotoxic carcinogen, and the side chain may be involved in the carcinogenicity of QdNOs. The further investigation is absolutely required to clarify the roles of side chain and metabolites of QdNOs in the carcinogenicity, and to reveal the exact molecular mechanisms for MEQ-induced carcinogenicity *in vivo*.

## Author Contributions

ZY conceived the idea. XW analyzed and discussed the data. QL analyzed and discussed the data and wrote the paper. ZL performed and revised the experiments. QL performed the experiments. SX and YP revised the paper. All authors discussed the results and contributed to the final manuscript.

## Conflict of Interest Statement

The authors declare that the research was conducted in the absence of any commercial or financial relationships that could be construed as a potential conflict of interest.

## Abbreviations

ALB: albumin
ALP: alkaline phosphatase
ALT: alanine aminotransferase
ANOVA: analysis of variance
AST: aspartate aminotransferase
b.w.: body weight
BIL: bilirubin
BUN: blood urea nitrogen
Ca: calcium
Cl^-^: chloride
CREA: creatinine
CRP: C-reactive protein
CT: coagulation time
GLU: glucose
HCT: hematocrit
H&E: hematoxylin and eosin
HGB: hemoglobin
IgM: immunoglobulin M
IgG: immunoglobulin
K^+^: potassium
KET: ketones
LEU: leukocytes
MCH: mean corpuscular hemoglobin
MCHC: mean corpuscular hemoglobin concentration
MCV: mean corpuscular volume
MPV: mean platelet volume
Na^+^: sodium
NIT: nitrite
P: inorganic phosphorus
PCT: plateletcrit
PDW: platelet distribution width
PLT: platelet count
PRO: protein
RBC: red blood cell count
RDW: red cell volume distribution
KM: Kun-Ming
SG: specific gravity
TBA: total bile acid
TCHO: total cholesterol
TG: triglyceride
TP: total protein
UA: uric acid
URE: urea
URO: urobilinogen
WBC: white blood cell count
