# Corrigendum: Mequindox Induced Genotoxicity and Carcinogenicity in Mice

**DOI:** 10.3389/fphar.2018.01387

**Published:** 2018-11-29

**Authors:** Qianying Liu, Zhixin Lei, Qin Wu, Deyu Huang, Shuyu Xie, Xu Wang, Yuanhu Pan, Zonghui Yuan

**Affiliations:** ^1^National Reference Laboratory of Veterinary Drug Residues (HZAU) and MAO Key Laboratory for Detection of Veterinary Drug Residues, Huazhong Agricultural University, Wuhan, China; ^2^Key Laboratory of Preventive Veterinary Medicine in Hubei Province, Huazhong Agricultural University, Wuhan, China; ^3^MOA Laboratory for Risk Assessment of Quality and Safety of Livestock and Poultry Products, Huazhong Agricultural University, Wuhan, China; ^4^Hubei Collaborative Innovation Center for Animal Nutrition and Feed Safety, Wuhan, China

**Keywords:** mequindox, quinoxaline, carcinogenicity, genotoxicity, KM mice

In the original article, there was a mistake in Figures 5B,F as published. The descriptions of Figures 5B,F did not match the figures displayed. The corrected Figure 5 appears below. The authors apologize for this error and state that this does not change the scientific conclusions of the article in any way. The original article has been updated.

**Figure 5:**
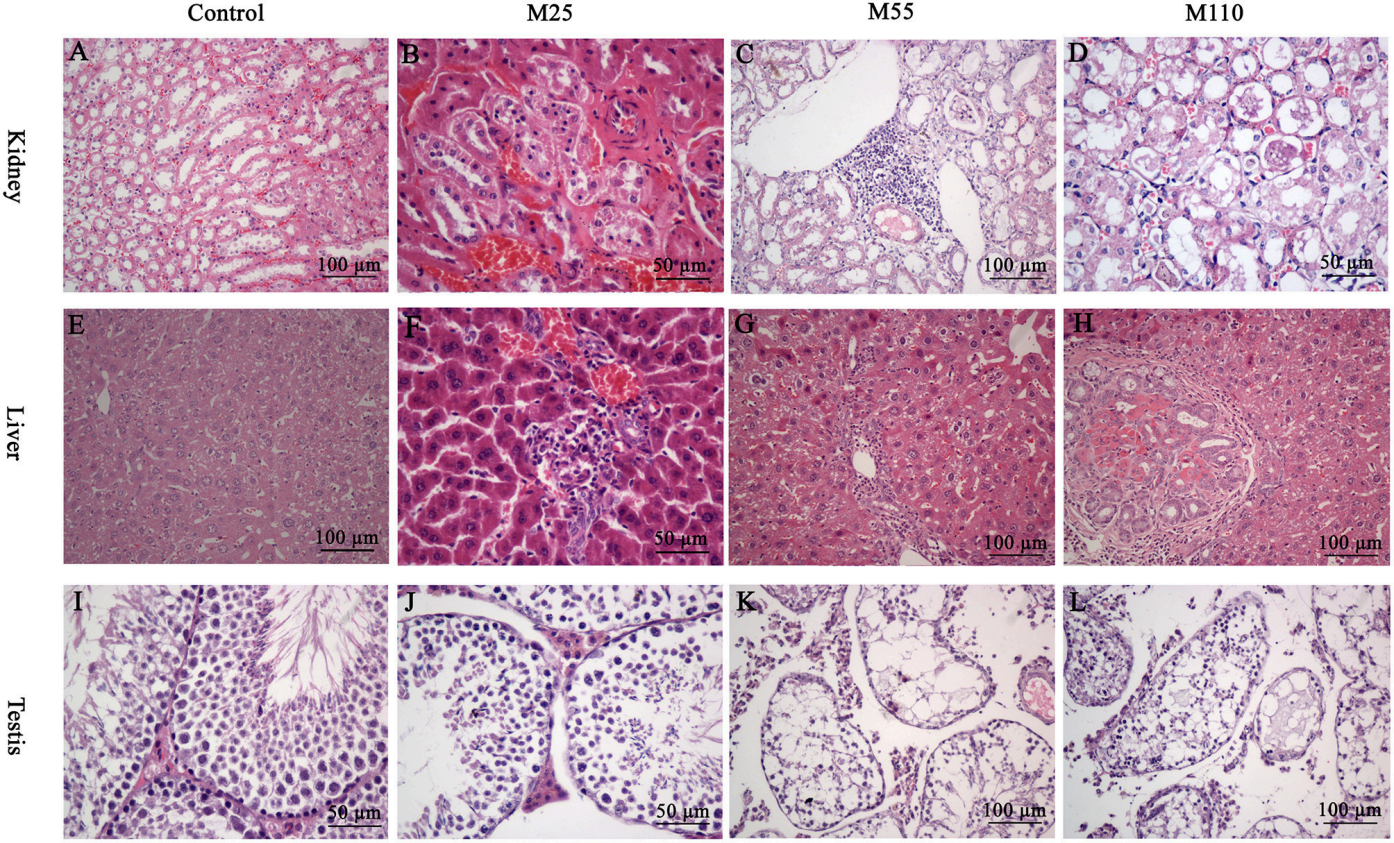
Selected microphotographs of kidney, liver and testis following dietary exposure to MEQ in the carcinogenicity tests (200×, 400×). **(A)** Kidney from control group (200×); **(B)** Kidney from the M25 mg/kg group showing kidney interstitial small blood vessels congestion, glomerular congestion (400×); **(C)** Kidney from the M55 mg/kg group showing aggregation of lymphocyte into a group around the central veins (200×); **(D)** Kidney from the M110 mg/kg group showing degeneration and necrosis of renal tubular epithelial cells (400×); **(E)** Liver from control group (200×); **(F)** Liver from the M25 mg/kg group showing degeneration and necrosis of hepatic cells (400×); **(G)** Liver from the M55 showing neutrophilic infiltrate within and around bile duct (200×); **(H)** Liver from the M110 mg/kg group showing proliferation in bile duct epithelium (200×); **(I)** Testis from the control group (400×); **(J)** Testis from the M25 mg/kg group showing a broadened testicular interstitium (400×); **(K)** Testis at the M55 mg/kg group showing an irregular arrangement as well as a decreased number of spermatogenic cells (200×); **(L)** Testis at the M110 mg/kg group showing necrosis of spermatogonia and spermatocytes in the lumen (200×).

## Conflict of interest statement

The authors declare that the research was conducted in the absence of any commercial or financial relationships that could be construed as a potential conflict of interest.

