# Supplementary material for: Mequindox Induced Genotoxicity and Carcinogenicity in Mice
Source: Front Pharmacol. 2018 Apr 10;9:361. doi: 10.3389/fphar.2018.00361 (PMC5902691; doi:10.3389/fphar.2018.00361)
Supplement: Supplementary file 1 [file Table_1.PDF]

Table 1S Statistical of death and pathological anatomy in mice during the carcinogenicity study.

	**Females**				**Males**			
	Control (n =9)	M25 (n = 6)	M55 (n = 5)	M110 (n = 5)	Control (n = 12)	M25 (n = 6)	M55 (n = 6)	M110 (n = 8)
17					AB			C
19	E					B		
22			A				AC	
23						A		A
25	A			A				
28					E			
31		A						
32								B
33					B			
35				C				
36			E					
37					E			
39	E					C		
40							E	E

42					AB			
43		B	E					
44								E
47	C				E			
48	A					C		
50					E			
51		A			B			
53								B
57			A		E		C	
60				C				
61	E							D
62						A		
67	D						B	
69			B		C			
70	E					B		
71		B		A				
72	A						B	
74					B			
75		C		B				C

77			C
78	A	C	

Note: M, mequindox; M25, 25 mg/kg diet; M55, 55 mg/kg diet; M110, 110 mg/kg diet.

A, Pneumonia; B, Hepatitis; C, Rupture of the spleen; D, Uterus suppuration; E, Enteritis.
