# Supplementary material for: Mequindox Induced Genotoxicity and Carcinogenicity in Mice
Source: Front Pharmacol. 2018 Apr 10;9:361. doi: 10.3389/fphar.2018.00361 (PMC5902691; doi:10.3389/fphar.2018.00361)
Supplement: Supplementary file 2 [file Table_2.PDF]

Table 2 Hematology parameters of mice fed mequindox at weeks 26 and 52 in carcinogenicity study (Mean±SD)

	**Females**				**Males**			
	Control (n = 5)	M25 (n = 5)	M55 (n = 5)	M110 (n = 5)	Control (n = 5)	M25 (n = 5)	M55 (n = 5)	M110 (n = 5)
**Week 26**								
WBC (10^9/L)	3.8 ± 1.1	4.9 ± 1.4	3.9 ± 1.2	4.8 ± 1.3	4.7 ± 1.2	5.3 ± 1.1	4.6 ± 1.3	4.9 ± 1.3
RBC (10^{12}/L)	4.1 ± 1.2	5.1 ± 0.9	5.7 ± 0.9**	5.3 ± 0.9*	5.09 ± 0.9	4.9 ± 0.9	4.6 ± 1.0	5.3 ± 0.7
HGB (g/L)	90.6 ± 11.6	96.0 ± 9.1	103.6 ± 10.7*	94.7 ± 8.5	91.0 ± 13.0	92.9 ± 8.9	86.2 ± 14.0	95.8 ± 10.8
HCT (%)	0.2 ± 0.07	0.2 ± 0.04	0.3 ± 0.04	0.2 ± 0.04	0.2 ± 0.03	0.2 ± 0.04	0.2 ± 0.05	0.2 ± 0.03
MCV (fl)	42.8 ± 0.9	44.0 ± 1.3*	43.6 ± 1.3	42.9 ± 1.6	44.5 ± 2.2	44.3 ± 1.6	46.5 ± 1.8	45.3 ± 2.4
MCH (Pg)	21.1 ± 4.3	19.1 ± 2.07	18.2 ± 1.1	18.8 ± 2.9	18.1 ± 0.9	19.1 ± 2.4	19.3 ± 1.9	18.2 ± 0.7
MCHC (g/L)	479.7 ± 74.4	419.0 ± 21.5	418.3 ± 27.0*	439.3 ± 85.6	408.2 ± 3.7	432.1 ± 58.4	411.9 ± 43.3	401.4 ± 19.7
RDW (%)	37.5 ± 1.5	33.7 ± 1.9**	33.6 ± 5.3*	34.1 ± 1.7**	30.6 ± 6.6	34.3 ± 3.6	26.1 ± 4.3	29.7 ± 6.7
PLT (10^9/L)	78.9 ± 66.2	60.8 ± 30.9	96.2 ± 24.6	149.9 ± 44.7*	36.0 ± 32.1	78.0 ± 43.8	170.8 ± 37.5	238.3 ± 80.2**
MPV (fl)	7.3 ± 0.6	7.4 ± 0.6	5.6 ± 1.2**	5.2± 0.7**	7.6 ± 0.5	5.8 ± 1.5	5.7 ± 1.2*	5.1 ± 0.3**
PCT (%)	0.07 ± 0.05	0.06 ± 0.04	0.1 ± 0.03	0.07 ± 0.02	0.1 ± 0.03	0.05 ± 0.03	0.1 ± 0.04	0.1 ± 0.05
PDW (%)	12.7 ± 1.1	12.6 ± 1.5	13.5 ± 1.3	14.5 ± 0.9**	11.7 ± 0.7	13.2 ± 1.1	14.2 ± 1.9	14.2 ± 0.6**
**Week 52**								
WBC (10^9/L)	4.2 ± 0.7	4.1 ± 0.6	3.9 ± 0.5	4.2 ± 0.8	4.2 ± 0.2	4.3 ± 0.8	4.2 ± 0.3	4.4 ± 0.7

RBC (10^{12}/L)	2.1 ± 0.3	2.5 ± 0.9	2.4 ± 0.7	6.2 ± 1.4**	2.4 ± 0.8	2.9 ± 0.9	2.9 ± 0.6	4.4 ± 0.6
HGB (g/L)	177.0 ± 43.6	174.0 ± 52.1	161.1 ± 22.5	293.6 ± 53.7*	144.5 ± 35.7	158.8 ± 46.9	161.7 ± 65.7	218.2 ± 30.1**
HCT (%)	0.09 ± 0.1	0.1 ± 0.02	0.1 ± 0.03	0.3 ± 0.06*	0.09 ± 0.02	0.1 ± 0.05	0.1 ± 0.04	0.2 ± 0.03
MCV (fl)	39.9 ± 2.5	41.6 ± 2.5	41.3 ± 1.2	43.7 ± 0.6*	41.6 ± 2.3	42.4 ± 2.7	42.2 ± 2.5	46.2 ± 3.9
MCH (Pg)	62.6 ± 10.9	69.6 ± 18.3	68.5 ± 15.6	48.2 ± 2.6*	60.2 ± 7.6	58.8 ± 13.7	57.9 ± 6.4	49.1 ± 2.2
MCHC (g/L)	28.7 ± 6.9	32.4 ± 3.7	36.6 ± 2.4*	37.1 ± 1.5*	34.9 ± 3.6	32.7 ± 4.3	29.3 ± 4.5	24.2 ± 8.1*
RDW (%)	65.4 ± 13.6	111.9 ± 95.6	89.8 ± 16.6	131.5 ± 46.6	200.0 ± 60.8	144.6 ± 15.1	122.0 ± 28.5	106.2 ± 16.3
PLT (10^9/L)	5.5 ± 1.2	6.6 ± 3.4	6.4 ± 1.03	5.1 ± 0.3	5.6 ± 1.04	6.03 ± 1.4	5.3 ± 0.6	6.3 ± 0.9
MPV (fl)	0.04 ± 0.03	0.07 ± 0.07	0.07 ± 0.04	0.06 ± 0.04	0.09 ± 0.04	0.08 ± 0.05	0.08 ± 0.05	0.07 ± 0.02
PCT (%)	14.1 ± 1.2	13.9 ± 1.1	13.3 ± 0.9	15.2 ± 1.8	15.1 ± 1.5	13.6 ± 1.9	14.1 ± 1.1	16.2 ± 1.1
PDW (%)	2.1 ± 0.6	2.5 ± 1.9	2.4 ± 0.7	6.2 ± 1.4**	2.4 ± 2.3	2.9 ± 0.9	2.9 ± 1.3	4.4 ± 0.5

Note: SD, standard deviation. M, mequindox; M25, 25 mg/kg diet; M55, 55 mg/kg diet; M110, 110 mg/kg diet. * Significantly different from control group at $p<0.05$.

** Significantly different from control group at $p<0.01$.
