# Supplementary material for: Mequindox Induced Genotoxicity and Carcinogenicity in Mice
Source: Front Pharmacol. 2018 Apr 10;9:361. doi: 10.3389/fphar.2018.00361 (PMC5902691; doi:10.3389/fphar.2018.00361)
Supplement: Supplementary file 3 [file Table_3.PDF]

Table 3S Serum clinical chemistry parameters of KM mice fed mequindox at weeks 26 and 52 in carcinogenicity study (Mean±SD)

	**Females**				**Males**			
	Control (n = 5)	M25 (n = 5)	M55 (n = 5)	M110 (n = 5)	Control (n = 5)	M25 (n = 5)	M55 (n = 5)	M110 (n = 5)
**Week 26**								
ALB (g/L)	30.7 ± 3.8	36.9 ± 3.0	38.4 ± 4.3	34.1 ± 6.4	41.9 ± 5.8	33.8 ± 3.6*	32.3 ± 4.3*	30.3 ± 4.1**
ALP (U/L)	50.6 ± 17.9	56.9 ± 15.1	63.7 ± 7.1	54.5 ± 13.8	54.3 ± 11.9	30.4 ± 8.1**	44.0 ± 13.5	37.0 ± 14.7
ALT (U/L)	89.4 ± 29.0	92.3 ± 29.4	93.6 ± 26.9	76.8 ± 24.1	97.5 ± 8.3	95.7 ± 8.5	125.3 ± 19.7*	84.7 ± 29.8
AST (U/L)	159.6 ± 19.3	151.1 ± 19.4	155.8 ± 12.7	137.1 ± 12.2	165.2 ± 29.0	178.0 ± 35.3	159.5 ± 21.0	144.6 ± 30.8
CREA (μmol/L)	18.2 ± 9.7	20.9 ± 11.1	18.1 ± 16.5	17.2 ± 11.6	19.3 ± 4.3	13.9 ± 5.2	9.8 ± 4.3**	17.3 ± 14.7
TG (mmol/L)	2.3 ± 0.6	2.2 ± 0.8	1.9 ± 0.5	1.9 ± 0.4	2.9 ± 0.9	3.1 ± 0.9	3.6 ±1.8	3.8 ± 1.2
Cl^- (mmol/L)	141.3 ± 4.9	135.9 ± 5.7*	137.5 ± 7.0	136.7 ± 6.1	135.3 ± 7.9	133.9 ± 4.3	138.0 ± 5.1	134.9 ± 6.5
URE (mmol/L)	4.7 ± 0.9	4.9 ± 1.3	4.5 ± 0.9	3.9 ± 0.5*	6.4 ± 3.9	4.0 ± 0.4	5.8 ± 1.9	3.9 ± 0.5
TBA (μmol/L)	2.9 ± 1.9	1.9 ± 1.3	2.2 ± 1.8	1.9 ± 0.9*	1.4 ± 1.4	1.5 ± 1.6	2.4 ± 1.7	1.0 ± 0.5
GLU (mmol/L)	6.5 ± 1.3	5.6 ± 1.7**	5.2 ± 0.4**	5.8 ± 0.8	7.8 ± 2.3	5.5 ± 1.2*	5.9 ± 1.4	7.4 ± 2.0
**Week 52**								
ALB (g/L)	25.3 ± 2.4	22.9 ± 3.7	22.2 ± 5.1	25.7 ± 1.7	19.5 ± 3.5	22.7 ± 4.5	23.5 ± 1.8*	20.32 ± 5.1
ALP (U/L)	122.0 ± 42.9	69.6 ± 12.1*	68.8 ± 19.3*	67.3 ± 15.8**	38.3 ± 5.6	50.7 ± 19.3	68.0 ± 26.0	27.4 ± 6.5*

ALT (U/L)	102.0 ± 6.5	87.0 ± 10.1**	94.3 ± 18.4	100.4 ± 9.9	94.4 ± 9.2	125.3 ± 14.0**	123.8 ± 7.7**	124.6 ± 25.4*
AST (U/L)	157.2 ± 18.2	162.0 ± 11.9	179.7 ± 21.2	185.0 ± 9.8**	181.8 ± 7.9	175.5 ± 10.1	189.8 ± 7.3	157.8 ± 9.2**
CREA (μmol/L)	144.8 ± 19.2	160.8 ± 42.6	162.3 ± 37.0	114.2 ± 48.2	153.9 ± 3.8	134.3 ± 30.5	123.1 ± 18.2**	96.2 ± 13.5**
LDHD (U/L)	713.6 ± 136.6	303.2 ± 67.2**	400.8 ± 61.7**	544.4 ± 133.3	535.4 ± 98.3	364.0 ± 120.50*	503.0 ± 121.7	594.8 ± 27.2
TG (mmol/L)	0.8 ± 0.2	0.7 ± 0.1	1.5 ± 0.8	0.8 ± 0.1	0.8 ± 0.2	1.8 ± 0.8*	1.9 ± 0.3**	1.0 ± 0.2
Ca^{++} (mmol/L)	3.4 ± 0.2	3.6 ± 0.2	3.4 ± 0.3*	3.4 ± 0.3*	3.5 ± 0.2	3.6 ± 0.2	3.6 ± 0.1	3.6 ± 0.4
Cl^{-} (mmol/L)	123.6 ± 2.9	124.8 ± 4.3	118.7 ± 7.2	123.8 ± 5.5	123.5 ± 2.5	125.4 ± 3.9	122.5 ± 3.9	125.9 ± 1.6
URE (mmol/L)	4.9 ± 0.4	5.5 ± 0.8	5.3 ± 0.2	5.1 ± 0.7	5.4 ± 0.3	5.7 ± 0.5	5.5 ± 1.0	5.0 ± 0.6
TCHO (mmol/L)	16.1 ± 0.6	12.1 ± 3.1**	12.8 ± 3.8	12.2 ± 1.2**	17.3 ± 0.3	15.6 ± 0.6**	15.7 ± 2.5*	13.1 ± 2.1**
UA (μmol/L)	343.3 ± 41.3	336.9 ± 65.0	338.3 ± 83.0	318.2 ± 49.5	240.6 ± 13.8	328.1 ± 26.3**	273.8 ± 39.2	371.5 ± 112.9**
TBA (μmol/L)	121.7 ± 8.6	119.9 ± 30.6	81.2 ± 23.1**	156.7 ± 13.1**	149.3 ± 9.6	140.6 ± 16.1	93.7 ± 21.2**	116.0 ± 5.2**
GGT (μmol/L)	26.5 ± 7.9	22.5 ± 2.9	20.0 ± 6.4	16.5 ± 3.2*	29.2 ± 1.9	14.4 ± 3.1**	21.4 ± 6.9*	21.4 ± 5.3*
GLU (mmol/L)	2.5 ± 1.2	3.9 ± 1.9	4.2 ± 0.6*	4.3 ± 0.7**	4.9 ± 0.3	5.5 ± 2.3	5.3 ± 1.9	5.0 ± 1.2
TP (g/L)	52.1 ± 6.9	62.6 ± 13.1	58.1 ± 15.1	59.9 ± 15.4	46.4 ± 2.2	65.5 ± 6.2**	59.7 ± 11.7*	43.4 ± 16.2

Note: SD, standard deviation. M, mequindox; M25, 25 mg/kg diet; M55, 55 mg/kg diet; M110, 110 mg/kg diet. * Significantly different from control group at $p<0.05$.

** Significantly different from control group at $p<0.01$.
