# Supplementary material for: Mequindox Induced Genotoxicity and Carcinogenicity in Mice
Source: Front Pharmacol. 2018 Apr 10;9:361. doi: 10.3389/fphar.2018.00361 (PMC5902691; doi:10.3389/fphar.2018.00361)
Supplement: Supplementary file 4 [file Table_4.PDF]

Table 4S Organ weights (g) in KM mice fed mequindox at weeks 26 and 52 in carcinogenicity study (Mean±SD)

	**Females**				**Males**			
	Control (n = 5)	M25 (n = 5)	M55 (n = 5)	M110 (n = 5)	Control (n = 5)	M25 (n = 5)	M55 (n = 5)	M110 (n = 5)
**Week 26**								
Final body weight (g)	36.3 ± 5.4	40.0 ± 7.6	41.4 ± 5.5	39.6 ± 9.1	49.3 ± 4.6	41.4 ± 4.6**	39.7 ± 2.7**	47.3 ± 5.7
Heart	0.19 ± 0.03	0.2 ± 0.02	0.2 ± 0.06	0.2 ± 0.03	0.3 ± 0.03	0.2 ± 0.04	0.2 ± 0.03**	0.2 ± 0.04
Liver	1.5 ± 0.2	1.5 ± 0.2	1.7 ± 0.3	1.5 ± 0.3	2.1 ± 0.08	2.0 ± 0.3	1.5 ± 0.6*	2.0 ± 0.3
Spleen	0.09 ± 0.02	0.08 ± 0.009	0.1 ± 0.02	0.09 ± 0.02	0.1 ± 0.03	0.07 ± 0.02*	0.1 ± 0.04	0.1 ± 0.03
Lungs	0.2 ± 0.02	0.2 ± 0.03	0.3 ± 0.09	0.26 ± 0.05	0.26 ± 0.04	0.2 ± 0.03	0.2 ± 0.03	0.3 ± 0.01
Kidney	0.4 ± 0.08	0.5 ± 0.08	0.4 ± 0.08	0.5 ± 0.1	0.6 ± 0.09	0.6 ± 0.05	0.5 ± 0.08	0.5 ± 0.113
Adrenal	0.01 ± 0.004	0.01 ± 0.006	0.01 ± 0.005	0.01 ± 0.005	0.02 ± 0.005	0.009 ± 0.003	0.009 ± 0.003	0.01 ± 0.003
Brain	0.4 ± 0.02	0.4 ± 0.04	0.4 ± 0.04	0.5 ± 0.1	0.5 ± 0.06	0.4 ± 0.02	0.5 ± 0.03	0.5 ± 0.03
Ovary	0.03 ± 0.008	0.02 ± 0.004*	0.1 ± 0.005	0.09 ± 0.01	-	-	-	-
Uterus	0.27 ± 0.1	0.2 ± 0.08	0.2 ± 0.1	0.3 ± 0.16	-	-	-	-
Testis	-	-	-	-	0.2 ± 0.07	0.3 ± 0.042	0.2 ± 0.03	0.3 ± 0.07
**Week 52**								
Final body weight (g)	42.0 ± 4.2	42.4 ± 3.3	40.1 ± 4.8	43.2 ± 5.3	56.4 ± 0.1	45.1 ± 5.1*	44.9 ± 5.9**	43.3 ± 4.7**

Heart	0.2 ± 0.03	0.2 ± 0.03	0.2 ± 0.03	0.2 ± 0.03	0.2 ± 0.006	0.2 ± 0.03	0.3 ± 0.05	0.2 ± 0.03
Liver	1.6 ± 0.2	1.6 ± 0.10	1.5 ± 0.2	1.7 ± 0.18	2.0 ± 0.14	2.1 ± 0.5	5.09 ± 7.7	1.8 ± 0.5
Spleen	0.07 ± 0.02	0.09 ± 0.01	0.08 ± 0.02	0.07 ± 0.02	0.1 ± 0.04	0.1 ± 0.02	0.1 ± 0.04	0.3 ± 0.04
Lungs	0.2 ± 0.03	0.2 ± 0.04	0.2 ± 0.05	0.3 ± 0.05	0.3 ± 0.03	0.3 ± 0.03	0.2 ± 0.06	0.3 ± 0.04
Kidney	0.4 ± 0.04	0.5 ± 0.04**	0.4 ± 0.07	0.4 ± 0.06	0.8 ± 0.09	0.7 ± 0.13	0.7 ± 0.13	0.7 ± 0.08
Adrenal	0.02 ± 0.006	0.02 ± 0.004	0.01 ± 0.002	0.01 ± 0.003	0.01 ± 0.006	0.01 ± 0.005	0.01 ± 0.006	0.01 ± 0.005
Brain	0.4 ± 0.04	0.5 ± 0.02	0.4 ± 0.02	0.4 ± 0.05	0.4 ± 0.02	0.4 ± 0.04	0.5 ± 0.03	0.5 ± 0.09
Ovary	0.06 ± 0.07	0.04 ± 0.01	0.03 ± 0.007	0.03 ± 0.005	-	-	-	-
Uterus	0.2 ± 0.2	0.2 ± 0.06	0.2 ± 0.08	0.2 ± 0.09	-	-	-	-
Testis	-	-	-	-	0.3 ± 0.07	0.3 ± 0.04	0.3 ± 0.04	0.2 ± 0.05

Note: SD = standard deviation, bw = body weight. M, mequindox; M25, 25 mg/kg diet; M55, 55 mg/kg diet; M110, 110 mg/kg diet.

* Significantly different from control group at $p<0.05$.

** Significantly different from control group at $p<0.01$.
