# Supplementary material for: Mequindox Induced Genotoxicity and Carcinogenicity in Mice
Source: Front Pharmacol. 2018 Apr 10;9:361. doi: 10.3389/fphar.2018.00361 (PMC5902691; doi:10.3389/fphar.2018.00361)
Supplement: Supplementary file 5 [file Table_5.PDF]

Table 5S Relative organ weights (mg/g final bw) in KM mice fed mequindox at weeks 26 and 52 in carcinogenicity study (Mean±SD)

	**Females**				**Males**			
	Control (n = 5)	M25 (n = 5)	M55 (n = 5)	M110 (n = 5)	Control (n = 5)	M25 (n = 5)	M55 (n = 5)	M110 (n = 5)
**Week 26**								
Final body weight (g)	36.3 ± 5.4	40.0 ± 7.6	41.4 ± 5.5	39.6 ± 9.1	49.3 ± 4.6	41.4 ± 4.6**	39.7 ± 2.7**	47.3 ± 5.7
Heart	4.6 ± 0.9	4.7 ± 0.6	4.9 ± 1.0	4.3 ± 0.5	5.1 ± 0.8	5.8 ± 1.2	5.0 ± 0.8	4.6 ± 0.8
Liver	37.1 ± 4.1	38.9 ± 3.4	39.2 ± 8.9	39.3 ± 4.5	43.3 ± 4.6	49.2 ± 8.8	41.7 ± 11.5	43.7 ± 8.5
Spleen	1.7 ± 0.4	2.0 ± 0.3	2.0 ± 0.5	1.6 ± 0.4	2.2 ± 0.7	1.8 ± 0.4	3. 5± 0.9*	2.6 ± 0.8
Lungs	5.5 ± 0.7	5.5 ± 0.9	5.8 ± 1.7	6.1 ± 1.4	5.4 ± 1.3	5.4 ± 0.6	5.5 ± 1.0	5.4 ± 1.7
Kidney	9.3 ± 1.4	10.6 ± 1.4	9.3 ± 2.2	8.6 ± 1.3	12.1 ± 2.7	13.8 ± 1.9	13.8 ± 2.4	10.3 ± 3.2
Adrenal[a]	0.4 ± 0.15	0.4 ± 0.13	0.4 ± 0.06	0.3 ± 0.09	0.2 ± 0.06	0.2 ± 0.05	0.2 ± 0.08	0.2 ± 0.08
Brain	10.2 ± 1.1	10.9 ± 1.2	11.1 ± 1.3	10.0 ± 1.7	9.2 ± 1.5	10.7 ± 1.5*	11.6 ± 1.1**	9.6 ± 1.2*
Ovary	1.3 ± 1.4	0.8 ± 0.2	0.8 ± 0.2	0.8 ± 0.1	-	-	-	-
Uterus	4.5 ± 2.1	4.5 ± 1.4	6.1 ± 2.2*	5.6 ± 2.0	-	-	-	-
Testis	-	-	-	-	4.8 ± 1.3	5.7 ± 1.3	6.0 ± 1.7*	5.7 ± 1.6
**Week 52**								
Final body weight (g)	42.0 ± 4.2	42.4 ± 3.3	40.1 ± 4.8	43.2 ± 5.3	56.4 ± 0.1	45.1 ± 5.1*	44.9 ± 5.9**	43.3 ± 4.7**

Heart	5.3 ± 0.9	5.0 ± 0.9	4.0 ± 1.4*	4.6 ± 1.6	4.2 ± 0.1	4.8 ± 0.6	5.6 ± 0.4	5.1 ± 0.7
Liver	41.1 ± 3.0	39.3 ± 6.7	39.9 ± 5.0	38.1 ± 11.9	35.8 ± 2.5	45.2 ± 5.9**	43.5 ± 4.5*	41.2 ± 11.2*
Spleen	2.5 ± 0.4	1.2 ± 0.4	2.4 ± 0.5	2.3 ± 0.9	2.4 ± 0.7	2.1 ± 0.3	2.1 ± 0.6	2.5 ± 1.4
Lungs	6.3 ± 1.1	5.7 ± 0.5	7.2 ± 1.7	6.7 ± 1.5	5.0 ± 0.6	5.6 ± 0.5	5.2 ± 1.6	6.3 ± 0.6
Kidney	11.9 ± 0.6	12.1 ± 1.2	10.8 ± 1.5	13.7 ± 2.1	13.5 ± 1.6	16.0 ± 2.1*	14.4 ± 0.9	14.1 ± 1.8
Adrenal[a]	0.35 ± 0.08	0.3 ± 0.1	0.3 ± 0.1	0.4 ± 0.1	0.2 ± 0.1	0.3 ± 0.8	0.3 ± 0.1	0.3 ± 0.08
Brain	12.3 ± 1.4	10.9 ± 2.1	10.7 ± 1.1	11.8 ± 2.9	7.8 ± 0.3	9.9 ± 0.9	10.1 ± 0.8	11.0 ± 1.1*
Ovary	0.9 ± 0.3	0.6 ± 0.2	0.7 ± 0.2	0.7 ± 0.4	-	-	-	-
Uterus	5.0 ± 2.4	4.8 ± 2.1	5.1 ± 2.4	2.8 ± 0.7**	-	-	-	-
Testis	-	-	-	-	4.1 ± 1.3	6.3 ± 1.1**	5.7 ± 0.8*	5.8 ± 1.5*

Note: SD = standard deviation, bw = body weight. M, mequindox; M25, 25 mg/kg diet; M55, 55 mg/kg diet; M110, 110 mg/kg diet.

[a] The unit of relative organ weights of Adrenal is g/10g.

* Significantly different from control group at $p<0.05$.

** Significantly different from control group at $p<0.01$.
